# Supplementary material for: Ecological drivers of sustained enzootic yellow fever virus transmission in Brazil, 2017–2021
Source: PLoS Negl Trop Dis. 2023 Jun 5;17(6):e0011407. doi: 10.1371/journal.pntd.0011407 (PMC10270639; doi:10.1371/journal.pntd.0011407)
Supplement: S1 Table — ID: identification of non-human primate carcasses. Area: sampling area. Urban-rural: urban-rural interface. RTqPCR-YFV: one-step real time polymerase chain reaction for investigation of yellow fever virus RNA, performed using methods described by Domingo and colleagues, 2012 (DOI: 10.1128/JCM.01799-12). Neg: negative result, pos: positive. * total RNA obtained from lung sample. (PDF) [file pntd.0011407.s001.pdf]

S1 Table: Non-human primate carcasses obtained from 2019 to 2021 and tested for the presence of yellow fever virus RNA

ID	Date	RTqPCR - YFV	Genus/family	area	Municipality	Mesoregion of Minas Gerais
914	09/01/19	neg	*Callithrix*	urban	Belo Horizonte	Metropolitana
915	09/01/19	neg	*Callithrix*	urban	Itabirito	Metropolitana
898	10/01/19	neg	*Callithrix*	rural	Moeda	Metropolitana
925	14/01/19	neg	*Callithrix*	urban	Belo Horizonte	Metropolitana
933	16/01/19	neg	Cebidae	urban	Pocos De Caldas	Sul/Sudoeste de Minas
931	22/01/19	neg	*Callithrix*	urban	Belo Horizonte	Metropolitana
935	30/01/19	neg	*Callithrix*	rural	Tocantins	Zona da Mata
927	05/02/19	neg	*Callithrix*	urban	Sao Francisco	Norte de Minas
926	06/02/19	neg	*Callithrix*	urban	Sete Lagoas	Metropolitana
929	06/02/19	neg	*Callithrix*	urban	Sete Lagoas	Metropolitana
922	11/02/19	neg	Cebidae	urban	Dionisio	Metropolitana
920	12/02/19	neg	*Callithrix*	urban	Belo Horizonte	Metropolitana
932	13/02/19	neg	Cebidae	urban-rural	Jacutinga	Sul/Sudoeste de Minas
934	25/02/19	neg	*Callithrix*	urban-rural	Cataguases	Zona da Mata
928	25/02/19	neg	*Callithrix*	urban	Leopoldina	Zona da Mata
921	26/02/19	neg	*Callithrix*	urban	Itabira	Metropolitana
924	28/02/19	neg	*Callithrix*	urban	Arceburgo	Sul/Sudoeste de Minas
923	28/02/19	neg	*Callithrix*	urban-rural	Porto Firme	Zona da Mata
940	19/03/19	neg	*Callithrix*	urban	Mateus Leme	Metropolitana
951	19/03/19	neg	*Callithrix*	rural	Mirabela	Norte de Minas
938	20/03/19	neg	*Callithrix*	urban	Belo Horizonte	Metropolitana
945	26/03/19	neg	*Callithrix*	rural	Arceburgo	Sul/Sudoeste de Minas

939	26/03/19	neg	*Callithrix*	rural	Piau	Zona da Mata
950	01/04/19	neg	*Callithrix*	urban	Belo Horizonte	Metropolitana
944	01/04/19	neg	*Callithrix*	urban	Pedras De Maria Da Cruz	Norte de Minas
937	05/04/19	neg	*Callithrix*	urban	Sao Tiago	Campo das Vertentes
956	10/04/19	neg	*Callithrix*	urban	Belo Horizonte	Metropolitana
954	10/04/19	neg	*Callithrix*	urban	Leopoldina	Zona da Mata
953	15/04/19	neg	*Callithrix*	urban	Visconde Do Rio Branco	Zona da Mata
942	17/04/19	neg	Cebidae	rural	Monte Santo De Minas	Sul/Sudoeste de Minas
949	23/04/19	neg	*Callithrix*	urban	Cataguases	Zona da Mata
936	24/04/19	neg	*Callithrix*	urban	Belo Horizonte	Metropolitana
947	24/04/19	neg	*Callithrix*	urban	Belo Horizonte	Metropolitana
946	25/04/19	neg	*Callithrix*	urban	Belo Horizonte	Metropolitana
962	10/05/19	neg	*Callithrix*	rural	Itauna	Triângulo/Alto Paranaíba
1090	11/05/2019	neg	*Callithrix*	urban	Matias Barbosa	Zona da Mata
1085	12/05/2019	neg	*Callithrix*	urban	Itamogi	Sul/Sudoeste de Minas
965	15/05/19	neg	*Callithrix*	urban	Curvelo	Central Mineira
957	22/05/19	neg	*Callithrix*	urban	Uberlandia	Triângulo/Alto Paranaíba
958	22/05/19	neg	*Callithrix*	urban	Uberlandia	Triângulo/Alto Paranaíba
959	22/05/19	neg	*Callithrix*	urban	Uberlandia	Triângulo/Alto Paranaíba
960	22/05/19	neg	*Callithrix*	urban	Uberlandia	Triângulo/Alto Paranaíba
968	22/05/19	neg	*Callithrix*	urban	Uberlandia	Triângulo/Alto Paranaíba
970	23/05/19	neg	*Callithrix*	urban	Belo Horizonte	Metropolitana
963	29/05/19	neg	*Callithrix*	urban	Curvelo	Central Mineira
967	29/05/19	neg	*Callithrix*	urban	Curvelo	Central Mineira
966	29/05/19	neg	*Callithrix*	urban	Nova Lima	Metropolitana

969	30/05/19	neg	*Callithrix*	urban	Leopoldina	Zona da Mata
1003	03/06/19	neg	*Callithrix*	urban	Belo Horizonte	Metropolitana
1007	05/06/19	neg	*Callithrix*	urban	Belo Horizonte	Metropolitana
996	06/06/19	neg	*Callithrix*	urban	Belo Horizonte	Metropolitana
1000	06/06/19	neg	*Callithrix*	rural	Paraopeba	Metropolitana
995	07/06/19	neg	*Callithrix*	urban	Ibirite	Triângulo/Alto Paranaíba
989	07/06/19	neg	*Callithrix*	urban	Lagoa Santa	Metropolitana
1001	07/06/19	neg	*Callithrix*	urban	Lagoa Santa	Metropolitana
1002	07/06/19	neg	*Callithrix*	urban	Lagoa Santa	Metropolitana
999	18/06/19	neg	*Callithrix*	urban	Conceicao Dos Ouros	Triângulo/Alto Paranaíba
987	18/06/19	neg	*Callithrix*	urban	Itabirito	Metropolitana
1004	19/06/19	neg	*Callithrix*	urban	Belo Horizonte	Metropolitana
1008	24/06/19	neg	*Callithrix*	urban-rural	Alem Paraiba	Zona da Mata
1005	24/06/19	neg	*Callithrix*	urban	Alem Paraiba	Zona da Mata
997	24/06/19	neg	*Callithrix*	urban	Leopoldina	Zona da Mata
991	26/06/19	neg	*Callithrix*	urban	Belo Horizonte	Metropolitana
1006	26/06/19	neg	*Callithrix*	urban	Nova Lima	Metropolitana
998	27/06/19	neg	*Callithrix*	urban	Belo Horizonte	Metropolitana
992	27/06/19	neg	*Callithrix*	urban	Itabira	Metropolitana
993	27/06/19	neg	*Callithrix*	urban	Timoteo	Vale do Rio Doce
984	01/07/19	neg	*Callithrix*	urban	Ubai	Norte de Minas
990	01/07/19	neg	*Callithrix*	urban	Ubai	Norte de Minas
994	01/07/19	neg	*Callithrix*	rural	Virgem Da Lapa	Jequitinhonha
980	02/07/19	neg	*Callithrix*	urban	Santa Luzia	Metropolitana
978	08/07/19	neg	*Callithrix*	rural	Alem Paraiba	Zona da Mata

986	08/07/19	neg	*Callithrix*	urban	Leopoldina	Zona da Mata
985	09/07/19	neg	*Callithrix*	rural	Sete Lagoas	Metropolitana
988	10/07/19	neg	*Callithrix*	urban	Teofilo Otoni	Vale do Mucuri
979	10/07/19	neg	*Callithrix*	rural	Uberlandia	Triângulo/Alto Paranaíba
983	10/07/19	neg	*Callithrix*	urban	Uberlandia	Triângulo/Alto Paranaíba
982	10/07/19	neg	Cebidae	rural	Piracema	Oeste de Minas
974	16/07/19	neg	*Callithrix*	urban	Santa Luzia	Metropolitana
975	23/07/19	neg	*Callithrix*	urban	Belo Horizonte	Metropolitana
976	24/07/19	neg	*Callithrix*	urban	Araguari	Triângulo/Alto Paranaíba
971	29/07/19	neg	*Callithrix*	urban	Formiga	Oeste de Minas
981	31/07/19	neg	*Callithrix*	urban	Araguari	Triângulo/Alto Paranaíba
972	31/07/19	neg	*Callithrix*	urban	Uberlandia	Triângulo/Alto Paranaíba
1034	05/08/2019	neg	*Callithrix*	urban	Cataguases	Zona da Mata
1036	05/08/2019	neg	*Callithrix*	urban	Cataguases	Zona da Mata
1026	08/08/2019	neg	*Callithrix*	urban	Sete Lagoas	Metropolitana
1027	08/08/2019	neg	*Callithrix*	urban	Sete Lagoas	Metropolitana
1031	09/08/2019	neg	*Callithrix*	urban-rural	Ibirite	Metropolitana
1098	11/08/2019	neg	*Callithrix*	urban	Belo Horizonte	Metropolitana
1025	12/08/2019	neg	Cebidae	rural	Piracema	Oeste de Minas
1024	26/08/2019	neg	*Callithrix*	urban	Leopoldina	Zona da Mata
1029	26/08/2019	neg	*Callithrix*	rural	Visconde Do Rio Branco	Zona da Mata
1018	28/08/2019	neg	*Callithrix*	rural	Guanhaes	Metropolitana
1009	28/08/2019	neg	*Callithrix*	urban	Guanhaes	Metropolitana
1011	28/08/2019	neg	*Callithrix*	urban	Santa Luzia	Metropolitana
1033	04/09/2019	neg	*Callithrix*	urban	Belo Horizonte	Metropolitana

1035	04/09/2019	neg	*Callithrix*	urban	Belo Horizonte	Metropolitana
1037	04/09/2019	neg	*Callithrix*	urban	Carangola	Metropolitana
1023	11/09/2019	neg	*Callithrix*	urban	Araxa	Metropolitana
1017	11/09/2019	neg	*Callithrix*	urban	Divinopolis	Metropolitana
1028	11/09/2019	neg	*Callithrix*	urban	Divinopolis	Metropolitana
1030	11/09/2019	neg	*Callithrix*	urban	Pedro Leopoldo	Metropolitana
1032	11/09/2019	neg	*Callithrix*	urban	Sao Francisco	Norte de Minas
1015	13/09/2019	neg	Cebidae	rural	Sao Sebastiao Do Oeste	Metropolitana
1021	17/09/2019	neg	*Callithrix*	rural	Bom Despacho	Metropolitana
1016	19/09/2019	neg	*Callithrix*	rural	Patrocinio	Triângulo/Alto Paranaíba
1014	30/09/2019	neg	*Callithrix*	urban	Leopoldina	Zona da Mata
1019	04/10/2019	neg	Cebidae	rural	Sabinopolis	Metropolitana
1010	16/10/2019	neg	*Callithrix*	urban	Patrocinio	Triângulo/Alto Paranaíba
1091	11/11/2019	neg	*Callithrix*	urban	Belo Horizonte	Metropolitana
1099	11/11/2019	neg	*Callithrix*	urban	Teofilo Otoni	Metropolitana
1081	05/12/2019	neg	*Callithrix*	urban	Araxa	Metropolitana
1094	13.01.2020	neg	*Callithrix*	urban	Belo Horizonte	Metropolitana
1096	13.01.2020	neg	*Callithrix*	urban	Belo Horizonte	Metropolitana
1101	13.01.2020	neg	*Callithrix*	urban	Belo Horizonte	Metropolitana
1107	13.01.2020	neg	*Callithrix*	urban	Belo Horizonte	Metropolitana
1116	05.02.2020	neg	*Callithrix*	rural	Patrocinio	Triângulo/Alto Paranaíba
1102	10.02.2020	neg	*Callithrix*	urban	Sao Jose Da Lapa	Metropolitana
1114	10.02.2020	neg	*Callithrix*	urban	Sao Jose Da Lapa	Metropolitana
1110	19.02.2020	neg	*Callithrix*	urban-rural	Belo Horizonte	Metropolitana
1113	02.03.2020	neg	Cebidae	rural	Itamarandiba	Jequitinhonha

1111	05.03.2020	neg	*Callithrix*	urban	Belo Horizonte	Metropolitana
1079	05.03.2020	neg	*Callithrix*	rural	Franciscopolis	Vale do Mucuri
1082	05.03.2020	neg	*Callithrix*	rural	Novo Cruzeiro	Jequitinhonha
1086	05.03.2020	neg	*Callithrix*	urban	Sao Tiago	Campo das Vertentes
1103	11.03.2020	neg	*Callithrix*	urban	Belo Horizonte	Metropolitana
1106	17.03.2020	neg	*Callithrix*	rural	Franciscopolis	Vale do Mucuri
1115	17.03.2020	neg	*Callithrix*	urban	Passos	Sul/Sudoeste de Minas
1105	15.04.2020	neg	*Callithrix*	urban	Belo Horizonte	Metropolitana
1108	20.04.2020	neg	*Callithrix*	urban	Igarape	Metropolitana
1112	06.05.2020	neg	*Callithrix*	rural	Campanha	Sul/Sudoeste de Minas
1104	07.05.2020	neg	*Callithrix*	urban	Belo Horizonte	Metropolitana
1063	12.06.2020	neg	*Callithrix*	urban	Belo Horizonte	Metropolitana
1057	16.06.2020	pos*	*Callithrix*	rural	Curvelo	Central Mineira
1039	17.06.2020	neg	*Callithrix*	urban	Itauna	Oeste de Minas
1074	18.06.2020	neg	*Callithrix*	urban	Ribeirao Das Neves	Metropolitana
1042	18.06.2020	neg	*Callithrix*	urban	Santa Luzia	Metropolitana
1056	22.06.2020	neg	*Callithrix*	urban	Ribeirao Das Neves	Metropolitana
1049	01.07.2020	neg	*Callithrix*	urban	Belo Horizonte	Metropolitana
1040	02.07.2020	neg	*Callithrix*	urban	Paracatu	Noroeste de Minas
1064	10.07.2020	neg	*Callithrix*	urban	Patrocinio	Triângulo/Alto Paranaíba
1071	17.07.2020	neg	*Callithrix*	urban	Belo Horizonte	Metropolitana
1075	23.07.2020	neg	*Callithrix*	urban	Entre Rios De Minas	Metropolitana
1048	04.08.2020	neg	*Callithrix*	urban	Uberaba	Triângulo/Alto Paranaíba
1076	07.08.2020	neg	*Callithrix*	urban	Curvelo	Central Mineira
1055	25.08.2020	neg	*Callithrix*	urban	Gov Valadares	Vale do Rio Doce

1050	25.08.2020	neg	*Callithrix*	urban	Sarzedo	Metropolitana
1066	04.09.2020	neg	*Aloutta*	urban-rural	Itapagipe	Metropolitana
1052	10.09.2020	neg	*Callithrix*	urban	Nova Lima	Metropolitana
1044	15.09.2020	neg	*Callithrix*	urban	Belo Horizonte	Metropolitana
1078	16.10.2020	neg	*Callithrix*	urban	Araguari	Triângulo/Alto Paranaíba
1065	05.11.2020	neg	*Callithrix*	urban	Belo Horizonte	Metropolitana
1118	01/02/2021	neg	*Callithrix*	urban	Itaúna	Oeste de Minas
1119	03/02/2021	neg	*Callithrix*	urban	Nova Lima	Metropolitana
1126	12/02/2021	neg	*Callithrix*	urban	Timóteo	Vale do Rio Doce
1140A	18/02/2021	neg	*Callithrix*	urban	Igaratinga	Oeste de Minas
1138	04/03/2021	neg	*Callithrix*	urban	Jaboticatubas	Metropolitana
1135	16/03/2021	neg	*Callithrix*	urban	Belo Horizonte	Metropolitana
1122	30/03/2021	neg	*Callithrix*	urban	Belo Horizonte	Metropolitana
1132	23/04/2021	neg	*Callithrix*	urban	Araguari	
1130	10/05/2021	neg	*Callithrix*	urban	Belo Horizonte	Metropolitana
1126	18/05/2021	neg	*Callithrix*	urban	Belo Horizonte	Metropolitana
1141	26/05/2021	neg	*Callithrix*	urban	Belo Horizonte	Metropolitana
1143	30/05/2021	neg	*Callithrix*	urban	Belo Horizonte	Metropolitana
1145	30/05/2021	neg	*Callithrix*	urban	Belo Horizonte	Metropolitana
1155	01/06/2021	neg	*Callithrix*	urban	Itapagipe	Metropolitana
1171	08/06/2021	neg	*Callithrix*	urban	Bom Despacho	Metropolitana
1149	17/06/2021	neg	*Callithrix*	urban	Belo Horizonte	Metropolitana
1175	17/06/2021	neg	*Callithrix*	urban	Belo Horizonte	Metropolitana
1151	23/06/2021	neg	*Callithrix*	urban	Belo Horizonte	Metropolitana
1153	23/06/2021	neg	*Callithrix*	urban	Belo Horizonte	Metropolitana

1147	25/06/2021	neg	*Callithrix*	urban	Belo Horizonte	Metropolitana
1159	01/07/2021	neg	*Callithrix*	urban	Belo Horizonte	Metropolitana
1167	01/07/2021	neg	*Callithrix*	urban	Ipatinga	Vale do Rio Doce
1181	12/07/2021	neg	*Callithrix*	urban	Belo Horizonte	Metropolitana
1095	13/11/2019	neg	*Callithrix*	rural	Carmo Do Rio Claro	Sul/Sudoeste de Minas
1092	17/11/2019	neg	*Callithrix*	rural	Brasilia De Minas	Norte de Minas
1080	19/11/2019	neg	*Callithrix*	urban	Araxa	Metropolitana
1083	21/11/2019	neg	*Callithrix*	urban	Belo Horizonte	Metropolitana
1087	23/10/2019	neg	*Callithrix*	urban	Sete Lagoas	Metropolitana
1097	23/10/2019	neg	Cebidae	rural	Munhoz	Sul/Sudoeste de Minas
1089	28/11/2019	neg	*Callithrix*	urban	Morada Nova De Minas	Central Mineira
1088	30/10/2019	neg	*Callithrix*	urban	Belo Horizonte	Metropolitana
1093	30/10/2019	neg	*Callithrix*	urban	Ibirite	Metropolitana
1084	31/12/2019	neg	*Callithrix*	rural	Bom Despacho	Metropolitana
1187	7/15/2021	neg	*Callithrix*	urban	Curvelo	Central Mineira

ID: identification of non-human primate carcasses. Area: sampling area. Urban-rural: urban-rural interface. RTqPCR-YFV: one-step real time polymerase chain reaction for investigation of yellow fever virus RNA, performed using methods described by Domingo and colleagues, 2012 (DOI: 10.1128/JCM.01799-12). Neg: negative result, pos: positive. * total RNA obtained from lung sample
