# Supplementary material for: Ecological drivers of sustained enzootic yellow fever virus transmission in Brazil, 2017–2021
Source: PLoS Negl Trop Dis. 2023 Jun 5;17(6):e0011407. doi: 10.1371/journal.pntd.0011407 (PMC10270639; doi:10.1371/journal.pntd.0011407)
Supplement: S2 Table — ID: identification of non-human primate carcasses. Epi week: epidemiological week. Area: sampling area. Urban-rural: urban-rural interface. Lat: latitude. Long: longitude. RTqPCR-YFV: one-step real time polymerase chain reaction for investigation of yellow fever virus RNA, performed using methods described by Domingo and colleagues, 2012 (DOI: 10.1128/JCM.01799-12). neg: negative result, pos: positive. * total RNA obtained from lung sample. Na: not available. (PDF) [file pntd.0011407.s002.pdf]

S2 Table: **Non-human primate carcasses used for analysis using generalized additive mixed models**

ID	date	RTqPCR - YFV		genus	period	area	carcass quality	Municipality	lat	long	Mesoregion of Minas Gerais
			Cq								
5	1/9/2017	pos	21.8	*Callithrix*	epi	rural	good	Ladainha	-17,63	-41,73	Vale do Mucuri
3	1/13/2017	pos	10.2	*Alouatta*	epi	rural	good	Jose Raydan	-18,21	-42,49	Vale do Rio Doce
6	1/13/2017	pos	34.5	*Callithrix*	epi	urban	good	Ipatinga	-19,46	-42,53	Vale do Rio Doce
4	1/13/2017	neg	na	*Callithrix*	epi	urban	good	Agua Boa	-17,99	-42,38	Vale do Rio Doce
7	1/17/2017	pos	17.8	*Callithrix*	epi	rural	intermediate	Sao Sebastiao do Maranhao	-18,08	-42,57	Vale do Rio Doce
1	1/19/2017	pos	15.3	*Callithrix*	epi	rural	good	Sabinopolis	-18,66	-43,08	Vale do Rio Doce
2	1/19/2017	neg	na	*Callithrix*	epi	urban	good	Ituiutaba	-18,96	-49,46	Triangulo/Alto Paranaíba
9	1/20/2017	pos	13.8	*Callithrix*	epi	rural	intermediate	Sao Joao Evangelista	-18,54	-42,76	Vale do Rio Doce
23	1/20/2017	pos	7.8	*Callicebus*	epi	rural	good	Sao Roque de Minas	-20,24	-46,36	Oeste de Minas
27	1/20/2017	pos	9.6	*Callicebus*	epi	rural	good	Ibia	-19,47	-46,53	Norte de Minas
10	1/23/2017	pos	17.5	*Callicebus*	epi	rural	good	Manhuacu	-20,25	-42,03	Zona da Mata
11	1/23/2017	neg	na	*Callithrix*	epi	rural	good	Sacramento	-19,86	-47,44	Triangulo/Alto Paranaíba
8	1/23/2017	neg	na	*Callithrix*	epi	urban	good	Belo Horizonte	-19,81	-43,95	Metropolitana
14	1/24/2017	neg	na	*Callithrix*	epi	rural	good	Curvelo	-18,75	-44,43	Central Mineira
49	1/25/2017	pos	10.5	*Alouatta*	epi	rural	good	Manhuacu	-20,25	-42,03	Zona da Mata
15	1/25/2017	pos	14.7	*Alouatta*	epi	rural	good	Chapada Gaucha	-15,3	-45,61	Norte de Minas
12	1/25/2017	pos	19.5	*Callicebus*	epi	rural	good	Luisburgo	-20,44	-42,1	Zona da Mata
13	1/25/2017	pos	30.8	*Callithrix*	epi	urban	good	Governador Valadares	-18,85	-41,94	Vale do Rio Doce
20	1/25/2017	neg	na	*Callithrix*	epi	urban	good	Contagem	-19,93	-44,05	Metropolitana
21	1/25/2017	neg	na	*Callithrix*	epi	urban	intermediate	Betim	-19,96	-44,19	Metropolitana
28	1/26/2017	pos	28.8	*Callicebus*	epi	rural	good	Japaraiba	-20,14	-45,5	Central Mineira

18	1/26/2017	pos	36.7	*Callithrix*	epi	urban	good	Curvelo	-18,75	-44,43	Central Mineira
16	1/27/2017	neg	na	*Callithrix*	epi	urban	good	Pocos de Caldas	-21,78	-46,56	Sul/Sudoeste de Minas
25	1/30/2017	pos	35	*Callithrix*	epi	urban	good	Belo Horizonte	-19,81	-43,95	Metropolitana
17	1/30/2017	neg	na	*Callithrix*	epi	urban-rural	good	Leopoldina	-21,53	-42,64	Zona da Mata
47	2/1/2017	pos	37	*Callicebus*	epi	rural	good	Sao Roque de Minas	-20,24	-46,36	Oeste de Minas
22	2/2/2017	pos	36.6	*Callithrix*	epi	urban	good	Nova Lima	-19,98	-43,84	Metropolitana
24	2/2/2017	neg	na	*Callithrix*	epi	urban	good	Ibia	-19,47	-46,53	Norte de Minas
26	2/3/2017	pos	14	*Callithrix*	epi	rural	intermediate	Chapada Gaucha	-15,3	-45,61	Norte de Minas
91	2/3/2017	pos	30.7	*Callicebus*	epi	urban-rural	good	Ouro Preto	-20,28	-43,5	Metropolitana
83	2/6/2017	pos	26	*Callicebus*	epi	rural	good	PIUMHI	-20,46	-45,95	Oeste de Minas
50	2/7/2017	neg	na	*Alouatta*	epi	rural	good	Sem Peixe	-20,1	-42,83	Zona da Mata
48	2/8/2017	pos	33	*Callithrix*	epi	rural	good	Sao Goncalo do Rio Abaixo	-19,82	-43,36	Metropolitana
84	2/8/2017	pos	32.3	*Callithrix*	epi	urban	intermediate	Ituiutaba	-18,96	-49,46	Triangulo/Alto Paranaíba
89	2/9/2017	pos	31.8	*Callithrix*	epi	urban	good	PIUMHI	-20,46	-45,95	Oeste de Minas
88	2/10/2017	pos	10.2	*Callithrix*	epi	rural	intermediate	Jose Raydan	-18,21	-42,49	Vale do Rio Doce
35	2/15/2017	pos	35.4	*Callithrix*	epi	rural	bad	Nova Resende	-21,12	-46,42	Sul/Sudoeste de Minas
34	2/15/2017	neg	na	*Callithrix*	epi	urban	bad	Vazante	-17,98	-46,9	Noroeste de Minas
38	2/16/2017	pos	35	*Callithrix*	epi	urban	good	Belo Horizonte	-19,81	-43,95	Metropolitana
40	2/16/2017	pos	25.5	*Callithrix*	epi	urban	good	Belo Horizonte	-19,81	-43,95	Metropolitana
46	2/16/2017	neg	na	*Callithrix*	epi	urban	bad	Betim	-19,96	-44,19	Metropolitana
45	2/16/2017	neg	na	*Callithrix*	epi	urban	bad	Belo Horizonte	-19,81	-43,95	Metropolitana
43	2/16/2017	neg	na	*Callithrix*	epi	urban	bad	Guape	-20,76	-45,91	Sul/Sudoeste de Minas
30	2/17/2017	pos	11.3	*Alouatta*	epi	urban	good	Santa Rita de Caldas	-22,02	-46,33	Sul/Sudoeste de Minas
37	2/17/2017	pos	33.7	*Callithrix*	epi	urban-rural	good	Campo Belo	-20,89	-45,27	Oeste de Minas

44	2/17/2017	pos	35.7	*Callithrix*	epi	rural	good	Alterosa	-21,24	-46,14	Sul/Sudoeste de Minas
29	2/17/2017	pos	8.5	*Callicebus*	epi	rural	good	Conceicao do Mato Dentro	-19,03	-43,42	Metropolitana
31	2/17/2017	pos	9.6	*Alouatta*	epi	urban	good	Santa Rita de Caldas	-22,02	-46,33	Sul/Sudoeste de Minas
33	2/17/2017	neg	na	*Callithrix*	epi	rural	bad	Martinho Campos	-19,33	-45,23	Central Mineira
155	2/17/2017	neg	na	*Callithrix*	epi	urban	bad	Morada Nova de Minas	-18,6	-45,35	Central Mineira
42	2/17/2017	neg	na	*Callithrix*	epi	urban	good	Pirapora	-17,34	-44,94	Norte de Minas
39	2/17/2017	neg	na	*Callithrix*	epi	urban	good	Barao de Cocais	-19,94	-43,48	Metropolitana
41	2/17/2017	neg	na	*Callithrix*	epi	urban-rural	good	Sarzedo	-20,03	-44,14	Metropolitana
36	2/17/2017	neg	na	*Callithrix*	epi	rural	intermediate	Carmo do Cajuru	-20,18	-44,77	Oeste de Minas
96	2/20/2017	pos	9.8	*Alouatta*	epi	rural	good	Abre Campo	-20,3	-42,47	Zona da Mata
80	2/21/2017	pos	12	*Callithrix*	epi	rural	good	Claraval	-20,39	-47,26	Sul/Sudoeste de Minas
73	2/21/2017	pos	35	*Callithrix*	epi	urban	intermediate	Uba	-21,12	-42,94	Zona da Mata
69	2/21/2017	pos	36	*Callithrix*	epi	urban	good	Betim	-19,96	-44,19	Metropolitana
85	2/21/2017	pos	11.5	*Callithrix*	epi	rural	good	Sao Jose da Barra	-20,71	-46,31	Sul/Sudoeste de Minas
72	2/21/2017	pos	29.1	*Callithrix*	epi	urban	bad	Ribeirao das Neves	-19,76	-44,08	Metropolitana
97	2/21/2017	pos	33.7	*Alouatta*	epi	rural	intermediate	Bom Jesus do Amparo	-19,7	-43,47	Metropolitana
78	2/21/2017	pos	9.5	*Callicebus*	epi	rural	good	Sao Joao Batista do Gloria	-20,64	-46,5	Sul/Sudoeste de Minas
79	2/21/2017	neg	na	*Callithrix*	epi	urban	bad	Governador Valadares	-18,85	-41,94	Vale do Rio Doce
71	2/21/2017	neg	na	*Callithrix*	epi	urban	bad	Lagoa da Prata	-20,02	-45,54	Central Mineira
70	2/21/2017	neg	na	*Callithrix*	epi	urban	good	Belo Horizonte	-19,81	-43,95	Metropolitana
74	2/22/2017	pos	29.5	*Callicebus*	epi	urban	good	Inconfidentes	-22,31	-46,32	Sul/Sudoeste de Minas
75	2/22/2017	pos	8.5	*Callicebus*	epi	urban	good	Aguanil	-20,94	-45,39	Oeste de Minas
81	2/24/2017	pos	32	*Callithrix*	epi	urban-rural	good	Santa Barbara	-19,95	-43,41	Metropolitana
76	2/24/2017	pos	10.3	*Callicebus*	epi	rural	good	Capitolio	-20,61	-46,05	Sul/Sudoeste de Minas

90	2/24/2017	pos	31.6	*Callithrix*	epi	urban	good	Ibirite	-20,02	-44,05	Metropolitana
86	2/24/2017	neg	na	*Callithrix*	epi	urban-rural	intermediate	Santa Luzia	-19,77	-43,85	Metropolitana
98	3/1/2017	pos	8.3	*Alouatta*	epi	rural	good	Campestre	-21,71	-46,24	Sul/Sudoeste de Minas
77	3/2/2017	pos	34.2	*Callicebus*	epi	rural	intermediate	Machado	-21,67	-45,92	Sul/Sudoeste de Minas
82	3/3/2017	pos	32.4	*Callithrix*	epi	urban	intermediate	Guape	-20,76	-45,91	Sul/Sudoeste de Minas
101	3/6/2017	pos	35	*Callithrix*	epi	rural	intermediate	Papagaios	-19,44	-44,74	Metropolitana
99	3/6/2017	pos	37	*Callithrix*	epi	urban	good	Felixlandia	-18,75	-44,89	Central Mineira
105	3/6/2017	neg	na	*Callithrix*	epi	urban	good	Belo Horizonte	-19,81	-43,95	Metropolitana
128	3/6/2017	neg	na	*Callithrix*	epi	urban-rural	good	Uba	-21,12	-42,94	Zona da Mata
104	3/6/2017	neg	na	*Callithrix*	epi	urban	intermediate	Betim	-19,96	-44,19	Metropolitana
132	3/7/2017	neg	na	*Callithrix*	epi	rural	good	Ibia	-19,47	-46,53	Norte de Minas
106	3/7/2017	neg	na	*Callithrix*	epi	urban	good	Ibia	-19,47	-46,53	Norte de Minas
127	3/7/2017	neg	na	*Callithrix*	epi	urban	good	Perdizes	-19,35	-47,29	Triangulo/Alto Paranaíba
130	3/7/2017	neg	na	*Callithrix*	epi	rural	intermediate	Carmo do Rio Claro	-20,97	-46,11	Sul/Sudoeste de Minas
111	3/8/2017	neg	na	*Callithrix*	epi	urban	bad	Ribeirao das Neves	-19,76	-44,08	Metropolitana
100	3/8/2017	neg	na	*Callithrix*	epi	rural	good	Ouro Branco	-20,52	-43,69	Metropolitana
129	3/8/2017	neg	na	*Callithrix*	epi	urban	intermediate	Alpinopolis	-20,86	-46,38	Sul/Sudoeste de Minas
131	3/8/2017	neg	na	*Callithrix*	epi	urban	intermediate	Ibirite	-20,02	-44,05	Metropolitana
124	3/9/2017	pos	36	*Callithrix*	epi	rural	good	Sao Goncalo do Rio Abaixo	-19,82	-43,36	Metropolitana
123	3/9/2017	neg	na	*Callithrix*	epi	urban	good	Congonhas	-20,5	-43,85	Metropolitana
168	3/10/2017	pos	30.2	na	epi	urban	good	Belo Horizonte	-19,81	-43,95	Metropolitana
116	3/10/2017	neg	na	*Callithrix*	epi	urban	good	Betim	-19,96	-44,19	Metropolitana
120	3/10/2017	neg	na	*Callithrix*	epi	urban-rural	good	Sao Goncalo do Rio Abaixo	-19,82	-43,36	Metropolitana
113	3/10/2017	neg	na	*Callithrix*	epi	urban-	good	Itaverava	-20,67	-43,61	Metropolitana

						rural					
117	3/13/2017	pos	36	*Callithrix*	epi	urban	intermediate	Mateus Leme	-19,98	-44,42	Metropolitana
121	3/13/2017	neg	na	*Callithrix*	epi	urban-rural	intermediate	Santa Luzia	-19,77	-43,85	Metropolitana
197	3/14/2017	pos	29	*Callithrix*	epi	rural	good	Visconde do Rio Branco	-21,01	-42,84	Zona da Mata
189	3/14/2017	pos	34	*Callithrix*	epi	urban	good	Nacip Raydan	-18,45	-42,24	Vale do Rio Doce
122	3/14/2017	pos	12.6	*Alouatta*	epi	urban	good	Senador Jose Bento	-22,16	-46,17	Sul/Sudoeste de Minas
118	3/14/2017	pos	33.5	*Callithrix*	epi	urban-rural	intermediate	Lagoa Santa	-19,62	-43,89	Metropolitana
167	3/14/2017	pos	7.9	*Callicebus*	epi	rural	intermediate	Fervedouro	-20,72	-42,27	Zona da Mata
190	3/14/2017	neg	na	*Callithrix*	epi	urban	bad	Governador Valadares	-18,85	-41,94	Vale do Rio Doce
244	3/14/2017	neg	na	*Callithrix*	epi	urban	good	Sabara	-19,88	-43,8	Metropolitana
217	3/14/2017	neg	na	*Callithrix*	epi	urban	intermediate	Governador Valadares	-18,85	-41,94	Vale do Rio Doce
239	3/14/2017	neg	na	*Callithrix*	epi	urban	intermediate	Governador Valadares	-18,85	-41,94	Vale do Rio Doce
56	3/15/2017	pos	8	*Callicebus*	epi	rural	intermediate	Andradas	-22,06	-46,56	Sul/Sudoeste de Minas
191	3/15/2017	pos	36	*Callithrix*	epi	rural	intermediate	Bonfinopolis de Minas	-16,56	-45,99	Noroeste de Minas
193	3/15/2017	pos	37	*Callithrix*	epi	rural	intermediate	Cabeceira Grande	-16,03	-47,09	Noroeste de Minas
141	3/15/2017	pos	14.3	*Alouatta*	epi	rural	good	Santa Rita de Caldas	-22,02	-46,33	Sul/Sudoeste de Minas
196	3/15/2017	pos	32.4	*Callithrix*	epi	urban	good	Alem Paraiba	-21,88	-42,7	Zona da Mata
60	3/16/2017	pos	37	*Callithrix*	epi	urban	good	Vicosa	-20,75	-42,88	Zona da Mata
156	3/16/2017	pos	36.5	*Callithrix*	epi	rural	intermediate	Senhora dos Remedios	-21,02	-43,58	Campo das Vertentes
59	3/16/2017	neg	na	*Callicebus*	epi	rural	good	Guimarania	-18,84	-46,79	Triangulo/Alto Paranaíba
53	3/16/2017	neg	na	*Callithrix*	epi	rural	good	Curvelo	-18,75	-44,43	Central Mineira
55	3/16/2017	neg	na	*Callithrix*	epi	rural	good	Goiana	-21,53	-43,2	Zona da Mata
164	3/16/2017	neg	na	*Callithrix*	epi	urban	good	Dores de Guanhaes	-19,05	-42,92	Vale do Rio Doce
54	3/16/2017	neg	na	*Callithrix*	epi	urban	good	Tocantins	-21,17	-43,01	Zona da Mata

57	3/16/2017	neg	na	*Callithrix*	epi	urban-rural	good	Brumadinho	-20,14	-44,2	Metropolitana
58	3/16/2017	neg	na	*Callithrix*	epi	urban-rural	good	Brumadinho	-20,14	-44,2	Metropolitana
61	3/16/2017	neg	na	*Callithrix*	epi	rural	intermediate	Romaria	-18,88	-47,58	Triangulo/Alto Paranaíba
62	3/16/2017	neg	na	*Callithrix*	epi	rural	intermediate	Uberlandia	-18,91	-48,27	Triangulo/Alto Paranaíba
52	3/16/2017	neg	na	*Callithrix*	epi	urban	intermediate	Curvelo	-18,75	-44,43	Central Mineira
215	3/17/2017	pos	33	*Callithrix*	epi	urban	good	Belo Horizonte	-19,81	-43,95	Metropolitana
157	3/17/2017	pos	33.4	*Callicebus*	epi	urban	na	Mateus Leme	-19,98	-44,42	Metropolitana
243	3/17/2017	neg	na	*Callithrix*	epi	rural	good	Sao Goncalo do Rio Abaixo	-19,82	-43,36	Metropolitana
158	3/17/2017	neg	na	*Callithrix*	epi	urban	good	Mario Campos	-20,05	-44,18	Metropolitana
152	3/17/2017	neg	na	*Callithrix*	epi	urban	intermediate	Itabirito	-20,25	-43,8	Metropolitana
159	3/17/2017	neg	na	*Callithrix*	epi	urban	intermediate	Sabara	-19,88	-43,8	Metropolitana
134	3/20/2017	pos	33	*Callithrix*	epi	rural	intermediate	Governador Valadares	-18,85	-41,94	Vale do Rio Doce
66	3/20/2017	pos	35	*Callithrix*	epi	rural	good	Santa Luzia	-19,77	-43,85	Metropolitana
64	3/20/2017	pos	33.5	*Callithrix*	epi	urban	good	Belo Horizonte	-19,81	-43,95	Metropolitana
63	3/20/2017	neg	na	*Callithrix*	epi	rural	good	Sete Lagoas	-19,46	-44,24	Metropolitana
133	3/20/2017	neg	na	*Callithrix*	epi	urban	good	Cataguases	-21,38	-42,69	Zona da Mata
140	3/20/2017	neg	na	*Alouatta*	epi	rural	intermediate	Marlieria	-19,71	-42,73	Vale do Rio Doce
65	3/20/2017	neg	na	*Callithrix*	epi	urban	intermediate	Belo Horizonte	-19,81	-43,95	Metropolitana
68	3/20/2017	neg	na	*Callithrix*	epi	urban	intermediate	Recreio	-21,52	-42,46	Zona da Mata
138	3/21/2017	pos	32	*Callithrix*	epi	urban	good	Ribeirao das Neves	-19,76	-44,08	Metropolitana
135	3/21/2017	pos	33.3	*Callithrix*	epi	urban	good	Belo Horizonte	-19,81	-43,95	Metropolitana
137	3/21/2017	neg	na	*Callithrix*	epi	urban	good	Itabirito	-20,25	-43,8	Metropolitana
269	3/22/2017	pos	28.6	*Callithrix*	epi	urban-rural	intermediate	Vespasiano	-19,69	-43,92	Metropolitana
246	3/22/2017	neg	na	*Callithrix*	epi	urban	bad	Araguari	-18,64	-48,18	Triangulo/Alto Paranaíba

257	3/22/2017	neg	na	*Callithrix*	epi	urban	bad	Corinto	-18,38	-44,45	Central Mineira
238	3/22/2017	neg	na	*Alouatta*	epi	rural	good	Borda da Mata	-22,27	-46,16	Sul/Sudoeste de Minas
259	3/22/2017	neg	na	*Callithrix*	epi	urban	good	Sao Joao Del Rei	-21,13	-44,26	Campo das Vertentes
249	3/22/2017	neg	na	*Callithrix*	epi	rural	intermediate	Paraopeba	-19,27	-44,4	Metropolitana
253	3/22/2017	neg	na	*Callithrix*	epi	urban	intermediate	Vespasiano	-19,69	-43,92	Metropolitana
204	3/23/2017	neg	na	*Callithrix*	epi	urban	good	Carmo do Rio Claro	-20,97	-46,11	Sul/Sudoeste de Minas
207	3/23/2017	neg	na	*Callithrix*	epi	urban	good	Sabara	-19,88	-43,8	Metropolitana
150	3/24/2017	pos	34	*Callithrix*	epi	urban-rural	intermediate	Santa Luzia	-19,77	-43,85	Metropolitana
143	3/24/2017	pos	36	*Callithrix*	epi	urban-rural	na	Belo Horizonte	-19,81	-43,95	Metropolitana
145	3/24/2017	neg	na	*Callithrix*	epi	urban	good	Muriae	-21,13	-42,36	Zona da Mata
144	3/24/2017	neg	na	*Callithrix*	epi	urban-rural	good	Belo Horizonte	-19,81	-43,95	Metropolitana
142	3/24/2017	neg	na	*Callithrix*	epi	urban-rural	good	Belo Horizonte	-19,81	-43,95	Metropolitana
149	3/24/2017	neg	na	*Callithrix*	epi	urban-rural	good	Inimutaba	-18,72	-44,36	Central Mineira
147	3/26/2017	pos	36.6	*Callithrix*	epi	urban	intermediate	Belo Horizonte	-19,81	-43,95	Metropolitana
148	3/26/2017	neg	na	*Callithrix*	epi	urban	intermediate	Belo Horizonte	-19,81	-43,95	Metropolitana
165	3/28/2017	neg	na	*Callicebus*	epi	rural	good	Capitolio	-20,61	-46,05	Sul/Sudoeste de Minas
154	3/28/2017	neg	na	*Callithrix*	epi	rural	good	Contagem	-19,93	-44,05	Metropolitana
219	3/28/2017	neg	na	*Callithrix*	epi	urban	good	Contagem	-19,93	-44,05	Metropolitana
146	3/28/2017	neg	na	*Callithrix*	epi	urban	good	Sarzedo	-20,03	-44,14	Metropolitana
245	3/29/2017	neg	na	*Callithrix*	epi	urban	good	Patrocinio	-18,94	-46,99	Triangulo/Alto Paranaíba
214	3/30/2017	pos	36	*Callithrix*	epi	urban	good	Mario Campos	-20,05	-44,18	Metropolitana
153	3/30/2017	neg	na	*Callithrix*	epi	rural	good	Felixlandia	-18,75	-44,89	Central Mineira
182	4/3/2017	pos	31	*Callithrix*	epi	urban-rural	good	Jaboticatubas	-19,51	-43,74	Metropolitana

172	4/3/2017	neg	na	*Callithrix*	epi	rural	bad	Morada Nova de Minas	-18,6	-45,35	Central Mineira
175	4/3/2017	neg	na	*Callithrix*	epi	urban-rural	good	Santa Luzia	-19,77	-43,85	Metropolitana
174	4/3/2017	neg	na	*Callicebus*	epi	rural	intermediate	Santo Antonio do Gama	-20,31	-42,6	Zona da Mata
170	4/3/2017	neg	na	*Callithrix*	epi	urban	intermediate	Contagem	-19,93	-44,05	Metropolitana
173	4/4/2017	neg	na	*Callithrix*	epi	urban	good	Ibirite	-20,02	-44,05	Metropolitana
405	4/4/2017	neg	na	*Callicebus*	epi	rural	intermediate	Pedro Teixeira	-21,7	-43,74	Zona da Mata
263	4/4/2017	neg	na	*Callithrix*	epi	urban-rural	intermediate	Visconde do Rio Branco	-21,01	-42,84	Zona da Mata
216	4/5/2017	pos	35	*Callithrix*	epi	urban	good	Lagoa da Prata	-20,02	-45,54	Central Mineira
161	4/5/2017	pos	10.2	*Alouatta*	epi	rural	good	Congonhal	-22,15	-46,03	Sul/Sudoeste de Minas
162	4/5/2017	pos	11.6	*Alouatta*	epi	rural	na	Bom Repouso	-22,47	-46,14	Sul/Sudoeste de Minas
169	4/5/2017	pos	36.9	*Callithrix*	epi	urban	na	Araguari	-18,64	-48,18	Triangulo/Alto Paranaíba
176	4/5/2017	pos	8.7	*Callicebus*	epi	rural	na	Sao Tiago	-20,91	-44,5	Campo das Vertentes
248	4/5/2017	neg	na	*Callithrix*	epi	urban	intermediate	Betim	-19,96	-44,19	Metropolitana
178	4/5/2017	neg	na	*Callicebus*	epi	rural	na	Andradas	-22,06	-46,56	Sul/Sudoeste de Minas
171	4/5/2017	neg	na	*Callithrix*	epi	urban	na	Uberlandia	-18,91	-48,27	Triangulo/Alto Paranaíba
180	4/5/2017	neg	na	*Callithrix*	epi	urban	na	Uberlandia	-18,91	-48,27	Triangulo/Alto Paranaíba
181	4/5/2017	neg	na	*Callithrix*	epi	urban	na	Uberlandia	-18,91	-48,27	Triangulo/Alto Paranaíba
177	4/5/2017	neg	na	*Callithrix*	epi	urban-rural	na	Betim	-19,96	-44,19	Metropolitana
179	4/6/2017	pos	26.5	*Callithrix*	epi	urban	good	Betim	-19,96	-44,19	Metropolitana
166	4/6/2017	pos	29.6	*Alouatta*	epi	urban	good	Alem Paraiba	-21,88	-42,7	Zona da Mata
240	4/6/2017	neg	na	*Callithrix*	epi	urban	bad	Santa Luzia	-19,77	-43,85	Metropolitana
241	4/6/2017	neg	na	*Callithrix*	epi	urban-rural	good	Pedro Leopoldo	-19,61	-44,04	Metropolitana
250	4/6/2017	neg	na	*Callithrix*	epi	urban	intermediate	Contagem	-19,93	-44,05	Metropolitana

183	4/6/2017	neg	na	*Callithrix*	epi	rural	na	Jampruca	-18,46	-41,8	Vale do Rio Doce
266	4/7/2017	pos	10.3	*Callithrix*	epi	urban	intermediate	Sao Tiago	-20,91	-44,5	Campo das Vertentes
265	4/7/2017	pos	9.7	*Callithrix*	epi	urban	intermediate	Sao Tiago	-20,91	-44,5	Campo das Vertentes
247	4/7/2017	neg	na	*Callithrix*	epi	urban-rural	intermediate	Augusto de Lima	-18,1	-44,26	Central Mineira
205	4/10/2017	neg	na	*Callithrix*	epi	rural	good	Jaboticatubas	-19,51	-43,74	Metropolitana
209	4/10/2017	neg	na	*Callithrix*	epi	urban	good	Sao Francisco	-15,94	-44,86	Norte de Minas
208	4/10/2017	neg	na	*Callithrix*	epi	urban	good	Sao Francisco	-15,94	-44,86	Norte de Minas
185	4/10/2017	neg	na	*Alouatta*	epi	rural	intermediate	Icarai de Minas	-16,21	-44,9	Norte de Minas
210	4/10/2017	neg	na	*Callithrix*	epi	rural	intermediate	Betim	-19,96	-44,19	Metropolitana
194	4/11/2017	pos	31	*Alouatta*	epi	rural	good	Congonhal	-22,15	-46,03	Sul/Sudoeste de Minas
268	4/11/2017	pos	32.7	*Callithrix*	epi	rural	intermediate	Claudio	-20,44	-44,76	Oeste de Minas
192	4/11/2017	pos	34.5	*Callithrix*	epi	rural	good	Itabirito	-20,25	-43,8	Metropolitana
313	4/11/2017	pos	35.5	*Callithrix*	epi	urban	good	Belo Horizonte	-19,81	-43,95	Metropolitana
186	4/11/2017	pos	8.9	*Alouatta*	epi	rural	good	Congonhal	-22,15	-46,03	Sul/Sudoeste de Minas
319	4/11/2017	neg	na	*Callithrix*	epi	urban	intermediate	Ituiutaba	-18,96	-49,46	Triangulo/Alto Paranaíba
213	4/12/2017	pos	10	*Callicebus*	epi	rural	good	Igaratinga	-19,95	-44,7	Oeste de Minas
310	4/12/2017	pos	35.9	*Callithrix*	epi	urban	bad	Nova Uniao	-19,69	-43,58	Metropolitana
308	4/12/2017	neg	na	*Callithrix*	epi	urban	bad	Belo Horizonte	-19,81	-43,95	Metropolitana
211	4/12/2017	neg	na	*Callithrix*	epi	urban	bad	Para de Minas	-19,86	-44,6	Metropolitana
203	4/12/2017	neg	na	*Callithrix*	epi	urban	good	Belo Horizonte	-19,81	-43,95	Metropolitana
202	4/12/2017	neg	na	*Callithrix*	epi	urban	intermediate	Divinopolis	-20,13	-44,88	Oeste de Minas
187	4/12/2017	neg	na	*Callithrix*	epi	urban	na	Divinopolis	-20,13	-44,88	Oeste de Minas
201	4/13/2017	pos	36.2	*Callithrix*	epi	urban-rural	intermediate	Itatiaiucu	-20,19	-44,42	Metropolitana
212	4/13/2017	neg	na	*Callithrix*	epi	rural	good	Rio Piracicaba	-19,92	-43,17	Metropolitana

267	4/17/2017	pos	32.1	*Callithrix*	epi	rural	intermediate	Vespasiano	-19,69	-43,92	Metropolitana
261	4/17/2017	neg	na	*Callithrix*	epi	urban	good	Brumadinho	-20,14	-44,2	Metropolitana
242	4/17/2017	neg	na	*Callithrix*	epi	urban	intermediate	Bom Despacho	-19,73	-45,25	Central Mineira
184	4/18/2017	pos	16.4	*Alouatta*	epi	rural	intermediate	Rio Doce	-20,24	-42,9	Zona da Mata
366	4/18/2017	neg	na	*Callithrix*	epi	urban	good	Sao Joaquim de Bicas	-20,04	-44,27	Metropolitana
315	4/18/2017	neg	na	*Callithrix*	epi	urban	good	Ribeirao das Neves	-19,76	-44,08	Metropolitana
188	4/19/2017	pos	13	*Alouatta*	epi	rural	intermediate	Congonhal	-22,15	-46,03	Sul/Sudoeste de Minas
236	4/19/2017	pos	13	*Alouatta*	epi	rural	intermediate	Congonhal	-22,15	-46,03	Sul/Sudoeste de Minas
274	4/19/2017	pos	15.2	*Alouatta*	epi	rural	good	Senador Amaral	-22,58	-46,17	Sul/Sudoeste de Minas
252	4/19/2017	neg	na	*Callithrix*	epi	urban	good	Belo Horizonte	-19,81	-43,95	Metropolitana
254	4/19/2017	neg	na	*Callithrix*	epi	urban	intermediate	Sete Lagoas	-19,46	-44,24	Metropolitana
314	4/20/2017	pos	33.8	*Callithrix*	epi	rural	bad	Nova Lima	-19,98	-43,84	Metropolitana
318	4/20/2017	neg	na	*Callithrix*	epi	rural	bad	Sao Domingos do Prata	-19,86	-42,96	Metropolitana
251	4/20/2017	neg	na	*Callithrix*	epi	rural	good	Cajuri	-20,79	-42,79	Zona da Mata
163	4/20/2017	neg	na	*Callithrix*	epi	urban	good	Belo Horizonte	-19,81	-43,95	Metropolitana
309	4/20/2017	neg	na	*Callithrix*	epi	urban	good	Nova Lima	-19,98	-43,84	Metropolitana
321	4/20/2017	neg	na	*Callithrix*	epi	urban	intermediate	Nova Lima	-19,98	-43,84	Metropolitana
200	4/24/2017	pos	36.5	*Callithrix*	epi	urban	bad	Ribeirao das Neves	-19,76	-44,08	Metropolitana
195	4/25/2017	pos	35	*Callithrix*	epi	urban-rural	good	Itabirito	-20,25	-43,8	Metropolitana
272	4/26/2017	neg	na	*Callicebus*	epi	rural	good	Coronel Xavier Chaves	-21,02	-44,22	Campo das Vertentes
230	4/26/2017	neg	na	*Callithrix*	epi	rural	good	Patrocinio de Muriae	-21,15	-42,21	Zona da Mata
221	4/26/2017	neg	na	*Callithrix*	epi	urban	good	Monte Carmelo	-18,72	-47,49	Triangulo/Alto Paranaíba
226	4/26/2017	neg	na	*Callithrix*	epi	urban	good	Sabara	-19,88	-43,8	Metropolitana
220	4/26/2017	neg	na	*Callithrix*	epi	rural	intermediate	Presidente Juscelino	-18,63	-44,05	Central Mineira

223	4/26/2017	neg	na	*Callithrix*	epi	urban	intermediate	Belo Horizonte	-19,81	-43,95	Metropolitana
222	4/26/2017	neg	na	*Callithrix*	epi	urban-rural	intermediate	Itajuba	-22,42	-45,45	Sul/Sudoeste de Minas
228	4/27/2017	neg	na	*Callithrix*	epi	urban	intermediate	Belo Oriente	-19,22	-42,48	Vale do Rio Doce
233	5/2/2017	neg	na	*Callithrix*	epi	rural	good	Nova Uniao	-19,69	-43,58	Metropolitana
232	5/2/2017	neg	na	*Callithrix*	epi	urban	good	Cataguases	-21,38	-42,69	Zona da Mata
275	5/2/2017	neg	na	*Callithrix*	epi	urban	good	Belo Horizonte	-19,81	-43,95	Metropolitana
227	5/3/2017	neg	na	*Callithrix*	epi	urban	good	Uberlandia	-18,91	-48,27	Triangulo/Alto Paranaíba
224	5/3/2017	neg	na	*Callithrix*	epi	urban	good	Belo Horizonte	-19,81	-43,95	Metropolitana
235	5/3/2017	neg	na	*Callithrix*	epi	urban	good	Sao Joao Del Rei	-21,13	-44,26	Campo das Vertentes
229	5/3/2017	neg	na	*Callithrix*	epi	urban	intermediate	Buenopolis	-17,87	-44,18	Central Mineira
237	5/5/2017	pos	11	*Alouatta*	epi	rural	intermediate	Rio Doce	-20,24	-42,9	Zona da Mata
225	5/5/2017	neg	na	*Callithrix*	epi	urban	intermediate	Belo Horizonte	-19,81	-43,95	Metropolitana
234	5/8/2017	pos	36.3	*Callithrix*	epi	rural	intermediate	Bom Jesus da Penha	-21,01	-46,52	Sul/Sudoeste de Minas
231	5/8/2017	neg	na	*Callithrix*	epi	urban	good	Cataguases	-21,38	-42,69	Zona da Mata
279	5/9/2017	pos	35.5	*Callithrix*	epi	urban	intermediate	Nova Lima	-19,98	-43,84	Metropolitana
294	5/9/2017	neg	na	*Callithrix*	epi	urban	good	Ribeirao das Neves	-19,76	-44,08	Metropolitana
271	5/9/2017	neg	na	*Alouatta*	epi	rural	intermediate	Santa Juliana	-19,3	-47,52	Triangulo/Alto Paranaíba
276	5/9/2017	neg	na	*Callithrix*	epi	urban	intermediate	Coronel Fabriciano	-19,51	-42,62	Vale do Rio Doce
277	5/9/2017	neg	na	*Callithrix*	epi	urban	intermediate	Governador Valadares	-18,85	-41,94	Vale do Rio Doce
293	5/9/2017	neg	na	*Callithrix*	epi	urban	na	Carneirinho	-19,69	-50,68	Triangulo/Alto Paranaíba
611	5/11/2017	neg	na	*Callithrix*	epi	urban	good	Belo Horizonte	-19,81	-43,95	Metropolitana
609	5/11/2017	neg	na	*Callithrix*	epi	urban	intermediate	Belo Horizonte	-19,81	-43,95	Metropolitana
612	5/12/2017	neg	na	*Callithrix*	epi	urban	good	Belo Horizonte	-19,81	-43,95	Metropolitana
258	5/12/2017	neg	na	*Callithrix*	epi	urban	good	Santa Luzia	-19,77	-43,85	Metropolitana

264	5/12/2017	neg	na	*Callithrix*	epi	urban	good	Santa Luzia	-19,77	-43,85	Metropolitana
255	5/15/2017	neg	na	*Callithrix*	epi	rural	good	Inhauma	-19,49	-44,39	Metropolitana
260	5/15/2017	neg	na	*Callithrix*	epi	urban	good	Nova Lima	-19,98	-43,84	Metropolitana
290	5/16/2017	pos	36	*Callithrix*	epi	urban-rural	intermediate	Natalandia	-16,5	-46,49	Noroeste de Minas
262	5/16/2017	neg	na	*Callithrix*	epi	urban	good	Sabara	-19,88	-43,8	Metropolitana
256	5/16/2017	neg	na	*Callithrix*	epi	urban	intermediate	Jequitiba	-19,23	-44,02	Metropolitana
291	5/17/2017	pos	29.5	*Callithrix*	epi	urban	na	Patos de Minas	-18,57	-46,51	Triangulo/Alto Paranaíba
608	5/23/2017	neg	na	*Callithrix*	epi	urban	intermediate	Brumadinho	-20,14	-44,2	Metropolitana
398	5/24/2017	pos	34.2	*Callithrix*	epi	urban	good	Belo Horizonte	-19,81	-43,95	Metropolitana
396	5/24/2017	neg	na	*Callithrix*	epi	urban	bad	Itajuba	-22,42	-45,45	Sul/Sudoeste de Minas
397	5/25/2017	pos	33.3	*Callithrix*	epi	urban	intermediate	Presidente Bernardes	-20,76	-43,18	Zona da Mata
402	5/26/2017	pos	33.8	*Callithrix*	epi	rural	na	Ouro Preto	-20,28	-43,5	Metropolitana
613	6/5/2017	neg	na	*Callithrix*	non-epi	urban	bad	Belo Horizonte	-19,81	-43,95	Metropolitana
403	6/5/2017	neg	na	*Callithrix*	non-epi	rural	good	Nova Lima	-19,98	-43,84	Metropolitana
401	6/5/2017	neg	na	*Callithrix*	non-epi	urban	good	Santa Luzia	-19,77	-43,85	Metropolitana
400	6/6/2017	pos	36.3	*Callithrix*	non-epi	urban	good	Cataguases	-21,38	-42,69	Zona da Mata
399	6/6/2017	neg	na	*Callithrix*	non-epi	urban	intermediate	Itajuba	-22,42	-45,45	Sul/Sudoeste de Minas
289	6/7/2017	neg	na	*Callithrix*	non-epi	urban	good	Uberlandia	-18,91	-48,27	Triangulo/Alto Paranaíba
287	6/7/2017	neg	na	*Callithrix*	non-epi	rural	intermediate	Sao Goncalo do Rio Preto	-18	-43,39	Jequitinhonha
286	6/7/2017	neg	na	*Callithrix*	non-epi	urban-rural	intermediate	Uberlandia	-18,91	-48,27	Triangulo/Alto Paranaíba
284	6/8/2017	neg	na	*Callithrix*	non-epi	rural	na	Santa Luzia	-19,77	-43,85	Metropolitana
283	6/13/2017	neg	na	*Callithrix*	non-epi	urban	na	Belo Horizonte	-19,81	-43,95	Metropolitana
282	6/14/2017	neg	na	*Callithrix*	non-epi	urban	bad	Nova Lima	-19,98	-43,84	Metropolitana
280	6/14/2017	neg	na	*Callithrix*	non-epi	urban	na	Itabirito	-20,25	-43,8	Metropolitana

281	6/20/2017	neg	na	*Callithrix*	non-epi	urban	bad	Fronteira	-20,26	-49,19	Triangulo/Alto Paranaíba
288	6/20/2017	neg	na	*Callithrix*	non-epi	urban	na	Governador Valadares	-18,85	-41,94	Vale do Rio Doce
292	6/20/2017	neg	na	*Callithrix*	non-epi	urban	na	Iturama	-19,72	-50,19	Triangulo/Alto Paranaíba
347	6/21/2017	pos	12	*Alouatta*	non-epi	rural	na	Prados	-21,05	-44,08	Campo das Vertentes
297	6/22/2017	neg	na	*Callithrix*	non-epi	urban	good	Belo Horizonte	-19,81	-43,95	Metropolitana
307	6/22/2017	neg	na	*Callithrix*	non-epi	urban	good	Uberlandia	-18,91	-48,27	Triangulo/Alto Paranaíba
306	6/22/2017	neg	na	*Callithrix*	non-epi	urban	intermediate	Tupaciguara	-18,59	-48,7	Triangulo/Alto Paranaíba
301	6/22/2017	neg	na	*Callithrix*	non-epi	urban	intermediate	Uberlandia	-18,91	-48,27	Triangulo/Alto Paranaíba
304	6/23/2017	neg	na	*Callithrix*	non-epi	na	good	DOM BOSCO	-16,65	-46,27	Noroeste de Minas
302	6/23/2017	neg	na	*Callithrix*	non-epi	urban	intermediate	Sabara	-19,88	-43,8	Metropolitana
303	6/26/2017	pos	35	*Callithrix*	non-epi	urban	good	Belo Horizonte	-19,81	-43,95	Metropolitana
316	6/27/2017	neg	na	*Callithrix*	non-epi	urban	good	Vespasiano	-19,69	-43,92	Metropolitana
317	6/27/2017	neg	na	*Callithrix*	non-epi	urban	intermediate	Vespasiano	-19,69	-43,92	Metropolitana
320	6/28/2017	pos	36.3	*Callithrix*	non-epi	urban	good	Uberlandia	-18,91	-48,27	Triangulo/Alto Paranaíba
312	6/28/2017	neg	na	*Callithrix*	non-epi	urban	good	Uberlandia	-18,91	-48,27	Triangulo/Alto Paranaíba
311	6/28/2017	neg	na	*Callithrix*	non-epi	urban	good	Itajuba	-22,42	-45,45	Sul/Sudoeste de Minas
298	6/29/2017	neg	na	*Callithrix*	non-epi	urban	good	Matias Barbosa	-21,86	-43,31	Zona da Mata
305	6/29/2017	neg	na	*Callithrix*	non-epi	urban	good	Matias Barbosa	-21,86	-43,31	Zona da Mata
323	7/1/2017	neg	na	*Callithrix*	non-epi	urban	na	Montes Claros	-16,73	-43,86	Norte de Minas
345	7/4/2017	pos	11.5	*Alouatta*	non-epi	rural	na	Sao Joao Del Rei	-21,13	-44,26	Campo das Vertentes
346	7/4/2017	neg	na	*Alouatta*	non-epi	rural	na	Sao Joao Del Rei	-21,13	-44,26	Campo das Vertentes
295	7/4/2017	neg	na	*Callithrix*	non-epi	urban-rural	na	Campos Gerais	-21,23	-45,75	Sul/Sudoeste de Minas
390	7/6/2017	pos	26	*Alouatta*	non-epi	rural	good	Alem Paraiba	-21,88	-42,7	Zona da Mata
389	7/6/2017	pos	20.9	*Alouatta*	non-epi	rural	good	Alem Paraiba	-21,88	-42,7	Zona da Mata

363	7/6/2017	neg	na	*Callithrix*	non-epi	urban	good	Acaiaca	-20,36	-43,14	Zona da Mata
360	7/7/2017	neg	na	*Callithrix*	non-epi	urban	good	Conselheiro Lafaiete	-20,66	-43,78	Metropolitana
361	7/13/2017	neg	na	*Callithrix*	non-epi	urban	good	Sete Lagoas	-19,46	-44,24	Metropolitana
348	7/13/2017	neg	na	*Callithrix*	non-epi	urban	na	Sete Lagoas	-19,46	-44,24	Metropolitana
349	7/13/2017	neg	na	*Callithrix*	non-epi	urban	na	Sete Lagoas	-19,46	-44,24	Metropolitana
353	7/14/2017	pos	34	*Callithrix*	non-epi	urban-rural	na	Contagem	-19,93	-44,05	Metropolitana
355	7/18/2017	pos	36.6	*Callithrix*	non-epi	urban	good	Teofilo Otoni	-17,85	-41,5	Vale do Mucuri
329	7/21/2017	neg	na	*Callithrix*	non-epi	rural	na	Pedro Leopoldo	-19,61	-44,04	Metropolitana
322	7/22/2017	neg	na	*Callithrix*	non-epi	urban	na	Montes Claros	-16,73	-43,86	Norte de Minas
336	7/24/2017	neg	na	*Callithrix*	non-epi	urban	na	Recreio	-21,52	-42,46	Zona da Mata
342	7/25/2017	neg	na	*Callithrix*	non-epi	urban	na	Santa Vitoria	-18,83	-50,12	Triangulo/Alto Paranaíba
324	7/25/2017	neg	na	*Callithrix*	non-epi	urban	na	Ibirite	-20,02	-44,05	Metropolitana
333	7/27/2017	neg	na	*Callithrix*	non-epi	urban	good	Belo Horizonte	-19,81	-43,95	Metropolitana
337	7/28/2017	neg	na	*Callithrix*	non-epi	urban	na	Belo Horizonte	-19,81	-43,95	Metropolitana
331	8/1/2017	neg	na	*Callithrix*	non-epi	urban	na	Belo Horizonte	-19,81	-43,95	Metropolitana
341	8/2/2017	neg	na	*Callicebus*	non-epi	rural	good	Frutal	-20,02	-48,94	Triangulo/Alto Paranaíba
338	8/2/2017	neg	na	*Callithrix*	non-epi	rural	na	Ouro Preto	-20,28	-43,5	Metropolitana
335	8/9/2017	neg	na	*Callithrix*	non-epi	rural	good	Moeda	-20,33	-44,05	Metropolitana
326	8/9/2017	neg	na	*Callithrix*	non-epi	urban	na	Uberlandia	-18,91	-48,27	Triangulo/Alto Paranaíba
384	8/18/2017	neg	na	*Callithrix*	non-epi	urban	bad	Nova Modica	-18,43	-41,5	Vale do Rio Doce
372	8/22/2017	neg	na	*Callithrix*	non-epi	urban	good	Nova Lima	-19,98	-43,84	Metropolitana
373	8/24/2017	neg	na	*Callithrix*	non-epi	urban	intermediate	Arceburgo	-21,36	-46,94	Sul/Sudoeste de Minas
404	8/30/2017	pos	35.7	*Callicebus*	non-epi	rural	good	Pedralva	-22,24	-45,46	Sul/Sudoeste de Minas
365	8/30/2017	pos	36.6	*Callithrix*	non-epi	urban	bad	Pirajuba	-19,9	-48,7	Triangulo/Alto Paranaíba

364	8/30/2017	neg	na	*Callithrix*	non-epi	rural	bad	Fronteira	-20,26	-49,19	Triangulo/Alto Paranaíba
374	9/4/2017	neg	na	*Callithrix*	non-epi	urban	good	Sao Roque de Minas	-20,24	-46,36	Oeste de Minas
371	9/4/2017	neg	na	*Callithrix*	non-epi	urban	intermediate	Belo Horizonte	-19,81	-43,95	Metropolitana
434	9/5/2017	pos	12.3	*Alouatta*	non-epi	rural	good	Lagoa Dourada	-20,91	-44,07	Campo das Vertentes
370	9/12/2017	neg	na	*Callithrix*	non-epi	urban	bad	Belo Horizonte	-19,81	-43,95	Metropolitana
383	9/13/2017	neg	na	*Callithrix*	non-epi	urban	good	Conceicao da Aparecida	-21,09	-46,2	Sul/Sudoeste de Minas
379	9/14/2017	neg	na	*Callithrix*	non-epi	urban	good	Belo Horizonte	-19,81	-43,95	Metropolitana
375	9/19/2017	neg	na	*Callithrix*	non-epi	rural	good	Fortuna de Minas	-19,56	-44,44	Metropolitana
388	9/20/2017	neg	na	*Callicebus*	non-epi	rural	good	Caldas	-21,92	-46,38	Sul/Sudoeste de Minas
386	9/20/2017	neg	na	*Callithrix*	non-epi	urban	intermediate	Sete Lagoas	-19,46	-44,24	Metropolitana
368	9/25/2017	neg	na	*Callithrix*	non-epi	urban	intermediate	Belo Horizonte	-19,81	-43,95	Metropolitana
367	9/25/2017	neg	na	*Callithrix*	non-epi	urban	intermediate	Alem Paraiba	-21,88	-42,7	Zona da Mata
381	9/26/2017	neg	na	*Callithrix*	non-epi	urban	good	Governador Valadares	-18,85	-41,94	Vale do Rio Doce
377	10/2/2017	neg	na	*Callithrix*	non-epi	rural	good	Canaa	-20,68	-42,62	Zona da Mata
376	10/10/2017	neg	na	*Callicebus*	non-epi	urban-rural	good	Extrema	-22,85	-46,31	Sul/Sudoeste de Minas
369	10/11/2017	neg	na	*Callithrix*	non-epi	urban	bad	Sabara	-19,88	-43,8	Metropolitana
378	10/16/2017	neg	na	*Callithrix*	non-epi	urban	good	Belo Horizonte	-19,81	-43,95	Metropolitana
382	10/16/2017	neg	na	*Callithrix*	non-epi	urban	good	Belo Horizonte	-19,81	-43,95	Metropolitana
380	10/16/2017	neg	na	*Callithrix*	non-epi	urban-rural	good	Nova Lima	-19,98	-43,84	Metropolitana
385	10/16/2017	neg	na	*Callicebus*	non-epi	urban	intermediate	Ouro Preto	-20,28	-43,5	Metropolitana
395	10/17/2017	pos	36.6	*Callicebus*	non-epi	rural	intermediate	Itapagipe	-19,9	-49,38	Triangulo/Alto Paranaíba
392	10/17/2017	neg	na	*Callithrix*	non-epi	urban	bad	Itapagipe	-19,9	-49,38	Triangulo/Alto Paranaíba
485	10/20/2017	pos	34	*Callithrix*	non-epi	urban	good	Sao Sebastiao do Paraiso	-20,91	-46,99	Sul/Sudoeste de Minas
408	10/26/2017	neg	na	*Callithrix*	non-epi	urban	intermediate	Igarape	-20,07	-44,3	Metropolitana

482	10/27/2017	neg	na	*Callithrix*	non-epi	rural	good	Varzea da Palma	-17,59	-44,73	Norte de Minas
430	10/30/2017	pos	14.5	*Alouatta*	non-epi	rural	good	Santana do Deserto	-21,95	-43,16	Zona da Mata
406	10/30/2017	pos	9.2	*Callithrix*	non-epi	urban-rural	good	Sabara	-19,88	-43,8	Metropolitana
407	10/30/2017	neg	na	*Callithrix*	non-epi	urban	good	Esmeraldas	-19,76	-44,31	Metropolitana
432	11/7/2017	pos	31.8	*Callicebus*	non-epi	rural	good	Sao Joao Batista do Gloria	-20,64	-46,5	Sul/Sudoeste de Minas
431	11/7/2017	pos	36.5	*Alouatta*	non-epi	rural	good	Sao Francisco	-15,94	-44,86	Oeste de Minas
614	11/7/2017	neg	na	*Callithrix*	non-epi	urban	good	Itamogi	-21,07	-47,04	Sul/Sudoeste de Minas
409	11/7/2017	neg	na	*Callithrix*	non-epi	urban	intermediate	Curvelo	-18,75	-44,43	Central Mineira
414	12/4/2017	pos	20.7	*Alouatta*	epi	rural	good	Madre de Deus de Minas	-21,48	-44,33	Campo das Vertentes
411	12/4/2017	neg	na	*Callithrix*	epi	urban	good	Sete Lagoas	-19,46	-44,24	Metropolitana
428	12/5/2017	neg	na	*Callithrix*	epi	urban-rural	intermediate	Sao Francisco	-15,94	-44,86	Norte de Minas
418	12/6/2017	pos	30.6	*Callithrix*	epi	urban	intermediate	Itajuba	-22,42	-45,45	Sul/Sudoeste de Minas
427	12/6/2017	pos	31.9	*Callithrix*	epi	urban	intermediate	Sabara	-19,88	-43,8	Metropolitana
417	12/6/2017	pos	35.2	*Callithrix*	epi	urban	good	Sabara	-19,88	-43,8	Metropolitana
425	12/6/2017	pos	35.3	*Callithrix*	epi	urban	intermediate	Sabara	-19,88	-43,8	Metropolitana
426	12/6/2017	pos	35.7	*Alouatta*	epi	rural	good	Bom Jesus do Galho	-19,82	-42,31	Vale do Rio Doce
422	12/6/2017	pos	35.8	*Callithrix*	epi	rural	good	Pirangucu	-22,52	-45,49	Sul/Sudoeste de Minas
415	12/6/2017	neg	na	*Callithrix*	epi	rural	good	Mariana	-20,37	-43,41	Metropolitana
419	12/6/2017	neg	na	*Callithrix*	epi	urban	good	Sabara	-19,88	-43,8	Metropolitana
429	12/7/2017	pos	34	*Callithrix*	epi	urban	good	Bela Vista de Minas	-19,83	-43,09	Metropolitana
423	12/7/2017	pos	37	*Callithrix*	epi	rural	intermediate	Juiz de Fora	-21,76	-43,35	Zona da Mata
421	12/7/2017	pos	15.3	*Alouatta*	epi	rural	good	Matias Barbosa	-21,86	-43,31	Zona da Mata
424	12/7/2017	pos	15.6	*Alouatta*	epi	urban-rural	good	Simao Pereira	-21,96	-43,31	Zona da Mata
416	12/11/2017	pos	33.5	*Callithrix*	epi	urban	intermediate	Belo Horizonte	-19,81	-43,95	Metropolitana

493	12/11/2017	neg	na	*Callithrix*	epi	urban	intermediate	Belo Horizonte	-19,81	-43,95	Metropolitana
420	12/12/2017	pos	33	*Callithrix*	epi	urban	good	Belo Horizonte	-19,81	-43,95	Metropolitana
412	12/12/2017	neg	na	*Callithrix*	epi	urban	good	Belo Horizonte	-19,81	-43,95	Metropolitana
616	12/12/2017	neg	na	*Callithrix*	epi	urban	good	Belo Horizonte	-19,81	-43,95	Metropolitana
413	12/12/2017	neg	na	*Callithrix*	epi	urban	intermediate	Juiz de Fora	-21,76	-43,35	Zona da Mata
440	12/13/2017	pos	15.2	*Alouatta*	epi	urban-rural	intermediate	Santo Antonio do Aventureiro	-21,75	-42,81	Zona da Mata
447	12/13/2017	pos	33.3	*Callithrix*	epi	urban	good	Contagem	-19,93	-44,05	Metropolitana
410	12/13/2017	neg	na	*Callithrix*	epi	urban	good	Iguatama	-20,17	-45,71	Oeste de Minas
442	12/18/2017	pos	28.7	*Callithrix*	epi	urban	good	Belo Horizonte	-19,81	-43,95	Metropolitana
448	12/19/2017	pos	11.7	*Alouatta*	epi	urban-rural	good	Juiz de Fora	-21,76	-43,35	Zona da Mata
435	12/19/2017	neg	na	*Callithrix*	epi	urban	good	Uberaba	-19,74	-47,93	Triangulo/Alto Paranaíba
449	12/20/2017	pos	27.6	*Callithrix*	epi	rural	good	Varzea da Palma	-17,59	-44,73	Norte de Minas
437	12/20/2017	neg	na	*Callithrix*	epi	urban	bad	Morada Nova de Minas	-18,6	-45,35	Central Mineira
441	12/21/2017	pos	30.3	*Callithrix*	epi	urban	good	Vespasiano	-19,69	-43,92	Metropolitana
443	12/26/2017	pos	16	*Alouatta*	epi	rural	good	Piau	-21,5	-43,32	Zona da Mata
439	12/27/2017	pos	11	na	epi	rural	good	Caete	-19,88	-43,67	Metropolitana
445	12/28/2017	pos	11	*Callithrix*	epi	urban	good	Belmiro Braga	-21,94	-43,41	Zona da Mata
436	12/28/2017	pos	35	*Callithrix*	epi	urban	good	Contagem	-19,93	-44,05	Metropolitana
446	12/28/2017	pos	28.5	*Callithrix*	epi	urban	good	Sabara	-19,88	-43,8	Metropolitana
444	12/29/2017	pos	29	*Callithrix*	epi	urban	intermediate	Vespasiano	-19,69	-43,92	Metropolitana
438	12/29/2017	neg	na	*Callithrix*	epi	na	bad	Belo Horizonte	-19,81	-43,95	Metropolitana
471	1/3/2018	pos	33	*Alouatta*	epi	rural	good	Dom Bosco	-16,65	-46,27	Noroeste de Minas
460	1/3/2018	pos	10.4	*Callithrix*	epi	urban	good	Nova Lima	-19,98	-43,84	Metropolitana
452	1/3/2018	pos	10.5	*Callithrix*	epi	rural	good	Rio Manso	-20,26	-44,3	Metropolitana

450	1/3/2018	pos	8.8	na	epi	rural	good	Nova Lima	-19,98	-43,84	Metropolitana
451	1/3/2018	neg	na	*Callithrix*	epi	urban	intermediate	Romaria	-18,88	-47,58	Triangulo/Alto Paranaíba
474	1/4/2018	pos	26.7	*Callithrix*	epi	urban	good	Belo Horizonte	-19,81	-43,95	Metropolitana
467	1/5/2018	pos	10	*Callithrix*	epi	rural	intermediate	Brumadinho	-20,14	-44,2	Metropolitana
463	1/5/2018	pos	11	*Alouatta*	epi	urban	good	Juiz de Fora	-21,76	-43,35	Zona da Mata
478	1/5/2018	pos	19.8	*Callithrix*	epi	urban	good	Santa Luzia	-19,77	-43,85	Metropolitana
461	1/5/2018	pos	33.2	na	epi	rural	good	Rio Novo	-21,45	-43,12	Zona da Mata
455	1/5/2018	pos	7.7	na	epi	rural	good	Itabirito	-20,25	-43,8	Metropolitana
472	1/5/2018	pos	9.3	*Callithrix*	epi	rural	good	Brumadinho	-20,14	-44,2	Metropolitana
479	1/5/2018	neg	na	*Callithrix*	epi	urban	good	Contagem	-19,93	-44,05	Metropolitana
453	1/8/2018	pos	10.5	*Alouatta*	epi	rural	good	Santa Barbara	-19,95	-43,41	Metropolitana
465	1/9/2018	pos	11	*Callithrix*	epi	rural	good	Piranga	-20,68	-43,3	Zona da Mata
475	1/9/2018	pos	11.1	na	epi	rural	good	Itatiaiucu	-20,19	-44,42	Metropolitana
468	1/9/2018	pos	34.1	*Callithrix*	epi	urban	intermediate	Carmo do Rio Claro	-20,97	-46,11	Sul/Sudoeste de Minas
481	1/10/2018	pos	14	*Callithrix*	epi	rural	intermediate	Brumadinho	-20,14	-44,2	Metropolitana
480	1/10/2018	pos	31	*Callithrix*	epi	urban-rural	good	Caete	-19,88	-43,67	Metropolitana
458	1/10/2018	pos	10.6	*Alouatta*	epi	rural	good	Lima Duarte	-21,84	-43,79	Zona da Mata
477	1/10/2018	pos	27.8	*Callithrix*	epi	urban	good	Betim	-19,96	-44,19	Metropolitana
454	1/11/2018	pos	30.3	*Callithrix*	epi	urban	good	Mateus Leme	-19,98	-44,42	Metropolitana
462	1/11/2018	pos	31.5	*Callithrix*	epi	urban	good	Esmeraldas	-19,76	-44,31	Metropolitana
456	1/15/2018	pos	14	*Callithrix*	epi	rural	good	Itaguara	-20,39	-44,48	Metropolitana
473/492	1/15/2018	pos	11.2	*Callithrix*	epi	rural	good	Sabara	-19,88	-43,8	Metropolitana
457/615	1/15/2018	pos	23.6	*Callithrix*	epi	urban	good	Belo Horizonte	-19,81	-43,95	Metropolitana
470/491	1/15/2018	pos	28.6	*Callithrix*	epi	urban	good	Vespasiano	-19,69	-43,92	Metropolitana

469/490	1/15/2018	pos	31.6	*Callithrix*	epi	urban	intermediate	Belo Horizonte	-19,81	-43,95	Metropolitana
466	1/15/2018	pos	32.6	*Callithrix*	epi	urban	good	Uba	-21,12	-42,94	Zona da Mata
476	1/15/2018	pos	9.5	*Callithrix*	epi	rural	good	Santa Barbara	-19,95	-43,41	Metropolitana
464	1/15/2018	pos	9.5	na	epi	rural	intermediate	Belo Vale	-20,4	-44,02	Metropolitana
500	1/16/2018	pos	33	*Callithrix*	epi	rural	intermediate	Esmeraldas	-19,76	-44,31	Metropolitana
497	1/16/2018	pos	34	*Callithrix*	epi	urban	good	Paracatu	-17,22	-46,87	Noroeste de Minas
508	1/16/2018	pos	10.3	*Callithrix*	epi	rural	good	Porto Firme	-20,67	-43,08	Zona da Mata
525	1/16/2018	pos	14.2	*Callithrix*	epi	rural	good	Bonfim	-20,32	-44,23	Metropolitana
502	1/16/2018	pos	32.2	*Callithrix*	epi	urban	good	Paracatu	-17,22	-46,87	Noroeste de Minas
538	1/16/2018	pos	33.9	*Callithrix*	epi	rural	intermediate	Chiador	-22	-43,05	Zona da Mata
524	1/16/2018	neg	na	*Callithrix*	epi	rural	good	Cataguases	-21,38	-42,69	Zona da Mata
495	1/17/2018	pos	10.2	na	epi	rural	good	Piranga	-20,68	-43,3	Zona da Mata
510	1/17/2018	pos	10.6	*Callithrix*	epi	rural	good	Brumadinho	-20,14	-44,2	Metropolitana
531	1/17/2018	pos	31.8	*Callithrix*	epi	urban	bad	Matozinhos	-19,55	-44,08	Metropolitana
537	1/17/2018	pos	35.5	*Callithrix*	epi	rural	good	Campos Gerais	-21,23	-45,75	Sul/Sudoeste de Minas
536	1/17/2018	pos	9.5	na	epi	rural	good	Presidente Bernardes	-20,76	-43,18	Zona da Mata
530	1/17/2018	neg	na	*Callithrix*	epi	urban	intermediate	Belo Horizonte	-19,81	-43,95	Metropolitana
515	1/18/2018	pos	10.8	na	epi	rural	intermediate	Itabirito	-20,25	-43,8	Metropolitana
516	1/18/2018	pos	11.5	*Callithrix*	epi	rural	intermediate	Bonfim	-20,32	-44,23	Metropolitana
503	1/18/2018	pos	28.4	*Callithrix*	epi	urban	good	Barao de Cocais	-19,94	-43,48	Metropolitana
505	1/18/2018	pos	36.5	*Callithrix*	epi	urban	intermediate	Goiana	-21,53	-43,2	Zona da Mata
534	1/18/2018	pos	9.7	*Callithrix*	epi	urban	good	Barao de Cocais	-19,94	-43,48	Metropolitana
533	1/18/2018	neg	na	*Callithrix*	epi	urban	good	Curvelo	-18,75	-44,43	Central Mineira
527	1/18/2018	neg	na	*Callithrix*	epi	rural	intermediate	Andrelandia	-21,74	-44,3	Sul/Sudoeste de Minas
514	1/18/2018	neg	na	*Callithrix*	epi	urban	intermediate	Buenopolis	-17,87	-44,18	Central Mineira

529	1/18/2018	neg	na	*Callithrix*	epi	urban	intermediate	Santos Dumont	-21,45	-43,55	Zona da Mata
496	1/19/2018	pos	36	*Callithrix*	epi	urban	good	Belo Horizonte	-19,81	-43,95	Metropolitana
522	1/19/2018	pos	14.1	*Alouatta*	epi	rural	good	Alvinopolis	-20,1	-43,04	Metropolitana
498	1/19/2018	pos	34.2	*Callithrix*	epi	urban	intermediate	Santa Barbara	-19,95	-43,41	Metropolitana
501	1/19/2018	pos	35.5	*Callithrix*	epi	urban	good	Belo Horizonte	-19,81	-43,95	Metropolitana
507	1/19/2018	neg	na	*Callithrix*	epi	urban	bad	Belo Horizonte	-19,81	-43,95	Metropolitana
519	1/22/2018	pos	30	*Callithrix*	epi	rural	intermediate	Santana de Cataguases	-21,28	-42,55	Zona da Mata
521	1/22/2018	pos	14.7	*Callithrix*	epi	rural	good	Moeda	-20,33	-44,05	Metropolitana
532	1/22/2018	pos	31.4	na	epi	rural	good	Rio Piracicaba	-19,92	-43,17	Metropolitana
528	1/22/2018	pos	8.9	*Callithrix*	epi	rural	intermediate	Porto Firme	-20,67	-43,08	Zona da Mata
511	1/22/2018	neg	na	*Callithrix*	epi	urban	good	Belo Horizonte	-19,81	-43,95	Metropolitana
582	1/23/2018	pos	8	na	epi	rural	intermediate	Ouro Preto	-20,28	-43,5	Metropolitana
509	1/23/2018	pos	9	*Callithrix*	epi	rural	intermediate	Brumadinho	-20,14	-44,2	Metropolitana
526	1/23/2018	pos	13	*Callithrix*	epi	urban	good	Lagoa Santa	-19,62	-43,89	Metropolitana
518	1/23/2018	pos	33	*Alouatta*	epi	rural	good	Uniao de Minas	-19,53	-50,33	Triangulo/Alto Paranaíba
535	1/23/2018	pos	11.8	*Callithrix*	epi	rural	bad	Brumadinho	-20,14	-44,2	Metropolitana
504	1/23/2018	pos	14.4	*Callithrix*	epi	rural	good	Nova Uniao	-19,69	-43,58	Metropolitana
517	1/23/2018	pos	18.7	*Callithrix*	epi	rural	good	Guaraciaba	-20,57	-43	Zona da Mata
583	1/23/2018	pos	30.1	*Callithrix*	epi	na	good	Confins	-19,63	-43,98	Metropolitana
588	1/23/2018	pos	35.4	*Callithrix*	epi	urban	intermediate	Sabara	-19,88	-43,8	Metropolitana
506	1/23/2018	neg	na	*Callithrix*	epi	na	intermediate	Itauna	-20,07	-44,57	Oeste de Minas
512	1/23/2018	neg	na	*Callithrix*	epi	rural	intermediate	Periquito	-19,15	-42,23	Vale do Rio Doce
586	1/24/2018	pos	11	*Alouatta*	epi	rural	good	Camanducaia	-22,75	-46,14	Sul/Sudoeste de Minas
592	1/24/2018	pos	11	na	epi	rural	good	Senhora de Oliveira	-20,79	-43,34	Zona da Mata
593	1/24/2018	pos	10.5	*Callithrix*	epi	rural	good	Nova Uniao	-19,69	-43,58	Metropolitana

591	1/24/2018	pos	24.7	*Callithrix*	epi	urban	good	Belo Horizonte	-19,81	-43,95	Metropolitana
605	1/25/2018	pos	8	na	epi	rural	good	Paula Candido	-20,87	-42,98	Zona da Mata
604	1/25/2018	pos	9	*Callithrix*	epi	rural	good	Senhora dos Remedios	-21,02	-43,58	Campo das Vertentes
560	1/25/2018	pos	11	na	epi	rural	good	Resende Costa	-20,92	-44,23	Campo das Vertentes
599	1/25/2018	pos	29	*Callithrix*	epi	rural	good	Sao Domingos do Prata	-19,86	-42,96	Metropolitana
563	1/25/2018	pos	30	*Callithrix*	epi	urban	intermediate	Lima Duarte	-21,84	-43,79	Zona da Mata
603	1/25/2018	pos	32	*Callithrix*	epi	urban	bad	Guaxupe	-21,3	-46,71	Sul/Sudoeste de Minas
600	1/25/2018	pos	10.3	*Callithrix*	epi	rural	good	Bom Jesus do Amparo	-19,7	-43,47	Metropolitana
584	1/25/2018	pos	10.4	*Callithrix*	epi	urban	intermediate	Joao Monlevade	-19,81	-43,17	Metropolitana
552	1/25/2018	pos	13.3	*Callithrix*	epi	na	intermediate	Itatiaiucu	-20,19	-44,42	Metropolitana
561	1/25/2018	pos	32.2	*Callithrix*	epi	urban-rural	good	Sete Lagoas	-19,46	-44,24	Metropolitana
580	1/25/2018	neg	na	*Callithrix*	epi	urban	intermediate	Divinopolis	-20,13	-44,88	Oeste de Minas
581	1/26/2018	pos	15	*Callithrix*	epi	urban	intermediate	Sarzedo	-20,03	-44,14	Metropolitana
601	1/26/2018	pos	10.8	*Callithrix*	epi	rural	good	Rio Piracicaba	-19,92	-43,17	Metropolitana
555	1/26/2018	pos	20.3	*Callithrix*	epi	rural	intermediate	Jeceaba	-20,53	-43,98	Metropolitana
607	1/26/2018	pos	27.3	*Callithrix*	epi	urban	intermediate	Vespasiano	-19,69	-43,92	Metropolitana
606	1/26/2018	pos	28.2	*Callithrix*	epi	urban	good	Santa Luzia	-19,77	-43,85	Metropolitana
558	1/26/2018	pos	36.4	*Callithrix*	epi	urban	bad	Curvelo	-18,75	-44,43	Central Mineira
554	1/26/2018	pos	9.2	*Callithrix*	epi	rural	intermediate	Rio Piracicaba	-19,92	-43,17	Metropolitana
556	1/26/2018	neg	na	*Callithrix*	epi	urban-rural	good	Santana do Paraiso	-19,36	-42,56	Vale do Rio Doce
578	1/29/2018	pos	9	na	epi	rural	intermediate	Ouro Preto	-20,28	-43,5	Metropolitana
566	1/29/2018	pos	13	*Callithrix*	epi	urban	intermediate	Nova Lima	-19,98	-43,84	Metropolitana
571	1/29/2018	pos	10.3	*Callithrix*	epi	rural	good	Itabirito	-20,25	-43,8	Metropolitana
548	1/29/2018	pos	13.6	*Callithrix*	epi	rural	good	Itaguara	-20,39	-44,48	Metropolitana

550	1/29/2018	pos	24.3	*Callithrix*	epi	urban	intermediate	Itabirito	-20,25	-43,8	Metropolitana
572	1/29/2018	pos	32.5	*Callithrix*	epi	urban	good	Belo Vale	-20,4	-44,02	Metropolitana
562	1/29/2018	pos	36.5	*Callithrix*	epi	urban	good	Belo Horizonte	-19,81	-43,95	Metropolitana
579	1/29/2018	neg	na	*Callithrix*	epi	urban	bad	Bambui	-20	-45,97	Oeste de Minas
574	1/29/2018	neg	na	*Callithrix*	epi	urban	good	Ponte Nova	-20,41	-42,9	Campo das Vertentes
565	1/29/2018	neg	na	*Callithrix*	epi	urban	good	Santa Luzia	-19,77	-43,85	Metropolitana
576	1/30/2018	pos	32	*Callithrix*	epi	urban	bad	Santa Luzia	-19,77	-43,85	Metropolitana
541	1/30/2018	pos	11.2	*Callithrix*	epi	rural	good	Santa Barbara	-19,95	-43,41	Metropolitana
564	1/30/2018	pos	11.3	*Callithrix*	epi	rural	intermediate	Olaria	-21,86	-43,93	Zona da Mata
549	1/30/2018	pos	11.8	*Callithrix*	epi	rural	intermediate	Coronel Pacheco	-21,58	-43,26	Zona da Mata
543	1/30/2018	pos	12.6	na	epi	rural	good	Itabirito	-20,25	-43,8	Metropolitana
553	1/30/2018	pos	9.9	na	epi	rural	good	Presidente Bernardes	-20,76	-43,18	Zona da Mata
567	1/30/2018	neg	na	na	epi	rural	bad	Campo Florido	-19,76	-48,57	Triangulo/Alto Paranaíba
570	1/30/2018	neg	na	*Callithrix*	epi	rural	good	Diamantina	-18,24	-43,6	Jequitinhonha
569	1/30/2018	neg	na	*Callithrix*	epi	urban	good	Ipatinga	-19,46	-42,53	Vale do Rio Doce
585	1/30/2018	neg	na	*Callithrix*	epi	urban	good	Jaboticatubas	-19,51	-43,74	Metropolitana
568	1/30/2018	neg	na	*Callithrix*	epi	rural	intermediate	Conquista	-19,93	-47,54	Triangulo/Alto Paranaíba
575	1/30/2018	neg	na	*Callithrix*	epi	urban	intermediate	Uberaba	-19,74	-47,93	Triangulo/Alto Paranaíba
577	1/30/2018	neg	na	*Callithrix*	epi	urban-rural	na	Esmeraldas	-19,76	-44,31	Metropolitana
540	1/31/2018	pos	10	na	epi	rural	good	Itatiaiucu	-20,19	-44,42	Metropolitana
544	1/31/2018	pos	14	*Callithrix*	epi	urban-rural	good	Nova Lima	-19,98	-43,84	Metropolitana
545	1/31/2018	pos	32	*Callithrix*	epi	urban	good	Astolfo Dutra	-21,31	-42,86	Zona da Mata
542	1/31/2018	pos	30.4	*Callithrix*	epi	urban	intermediate	Leopoldina	-21,53	-42,64	Zona da Mata
546	1/31/2018	pos	33.5	*Callithrix*	epi	urban	bad	Cataguases	-21,38	-42,69	Zona da Mata

587	1/31/2018	pos	9.5	na	epi	rural	intermediate	Congonhal	-22,15	-46,03	Sul/Sudoeste de Minas
557	1/31/2018	neg	na	*Callithrix*	epi	rural	bad	Leopoldina	-21,53	-42,64	Zona da Mata
551	1/31/2018	neg	na	na	epi	urban	good	Pocos de Caldas	-21,78	-46,56	Sul/Sudoeste de Minas
547	1/31/2018	neg	na	*Callithrix*	epi	urban	intermediate	Cataguases	-21,38	-42,69	Zona da Mata
657	2/1/2018	pos	14	*Callithrix*	epi	urban	good	Itabirito	-20,25	-43,8	Metropolitana
635	2/1/2018	pos	33	*Callithrix*	epi	urban	intermediate	Lagoa Santa	-19,62	-43,89	Metropolitana
656	2/1/2018	pos	15.1	*Callithrix*	epi	urban	intermediate	Nova Lima	-19,98	-43,84	Metropolitana
661	2/1/2018	pos	26.6	*Callithrix*	epi	urban	intermediate	Rio Piracicaba	-19,92	-43,17	Metropolitana
621	2/1/2018	pos	36.2	*Callithrix*	epi	urban	intermediate	Contagem	-19,93	-44,05	Metropolitana
636	2/2/2018	pos	11	*Callithrix*	epi	rural	intermediate	Ouro Preto	-20,28	-43,5	Metropolitana
639	2/2/2018	pos	24.5	*Callithrix*	epi	urban	good	Ipatinga	-19,46	-42,53	Vale do Rio Doce
629	2/2/2018	pos	31.5	*Callithrix*	epi	rural	intermediate	Porto Firme	-20,67	-43,08	Zona da Mata
638	2/2/2018	pos	8.4	*Callithrix*	epi	rural	good	Alto Rio Doce	-21,02	-43,41	Zona da Mata
637	2/5/2018	pos	8.9	*Callithrix*	epi	urban	good	Itabirito	-20,25	-43,8	Metropolitana
650	2/5/2018	pos	9.2	na	epi	rural	good	Nova Lima	-19,98	-43,84	Metropolitana
660	2/6/2018	pos	11.2	*Callithrix*	epi	rural	bad	Alfredo Vasconcelos	-21,14	-43,77	Campo das Vertentes
662	2/6/2018	pos	28.7	*Callithrix*	epi	rural	bad	Desterro de Entre Rios	-20,66	-44,33	Metropolitana
654	2/6/2018	pos	8.2	na	epi	rural	good	Ouro Branco	-20,52	-43,69	Metropolitana
655	2/7/2018	pos	9	na	epi	rural	good	Sapucai Mirim	-22,74	-45,74	Sul/Sudoeste de Minas
641	2/7/2018	pos	32.7	*Callithrix*	epi	rural	intermediate	Tocos do Moji	-22,37	-46,09	Sul/Sudoeste de Minas
631	2/7/2018	pos	7.6	na	epi	urban	good	Santana do Paraiso	-19,36	-42,56	Vale do Rio Doce
632	2/7/2018	neg	na	na	epi	rural	good	Conceicao das Pedras	-22,16	-45,45	Sul/Sudoeste de Minas
619	2/7/2018	neg	na	*Callithrix*	epi	urban	good	Monte Carmelo	-18,72	-47,49	Triangulo/Alto Paranaíba
630	2/7/2018	neg	na	*Callithrix*	epi	urban	good	Papagaios	-19,44	-44,74	Metropolitana
643	2/8/2018	pos	33	*Callithrix*	epi	urban	intermediate	Sete Lagoas	-19,46	-44,24	Metropolitana

633	2/8/2018	pos	10.4	na	epi	rural	good	Carmo do Cajuru	-20,18	-44,77	Oeste de Minas
652	2/8/2018	pos	30.4	*Callithrix*	epi	urban	good	Sete Lagoas	-19,46	-44,24	Metropolitana
634	2/8/2018	pos	33.2	*Callithrix*	epi	rural	good	Sete Lagoas	-19,46	-44,24	Metropolitana
653	2/9/2018	pos	30.2	*Callithrix*	epi	urban	bad	Contagem	-19,93	-44,05	Metropolitana
659	2/9/2018	neg	na	*Callithrix*	epi	rural	good	Pedrinopolis	-19,22	-47,46	Triangulo/Alto Paranaíba
647	2/15/2018	pos	33	*Callithrix*	epi	urban	good	Senador Firmino	-20,91	-43,09	Zona da Mata
622	2/15/2018	pos	36	*Callithrix*	epi	urban	intermediate	Nova Era	-19,75	-43,03	Metropolitana
620	2/15/2018	neg	na	*Callithrix*	epi	urban	good	Santana dos Montes	-20,78	-43,69	Metropolitana
626	2/15/2018	neg	na	*Callithrix*	epi	rural	intermediate	Presidente Juscelino	-18,63	-44,05	Central Mineira
640	2/16/2018	pos	31	*Callithrix*	epi	urban	intermediate	Divinopolis	-20,13	-44,88	Oeste de Minas
645	2/16/2018	pos	34	*Callithrix*	epi	urban	intermediate	Divinopolis	-20,13	-44,88	Oeste de Minas
642	2/16/2018	pos	36	*Callithrix*	epi	urban	good	Santa Luzia	-19,77	-43,85	Metropolitana
651	2/16/2018	pos	30.2	*Callithrix*	epi	urban	good	Belo Horizonte	-19,81	-43,95	Metropolitana
649	2/16/2018	pos	34.6	*Callithrix*	epi	urban	good	Visconde do Rio Branco	-21,01	-42,84	Zona da Mata
624	2/16/2018	pos	36.7	*Callithrix*	epi	urban	intermediate	Muzambinho	-21,37	-46,52	Sul/Sudoeste de Minas
628	2/16/2018	neg	na	*Callithrix*	epi	urban-rural	intermediate	Ribeirao das Neves	-19,76	-44,08	Metropolitana
678	2/19/2018	pos	36	*Callithrix*	epi	urban	good	Uberlandia	-18,91	-48,27	Triangulo/Alto Paranaíba
625	2/19/2018	pos	33.8	*Callithrix*	epi	urban	intermediate	Belo Horizonte	-19,81	-43,95	Metropolitana
673	2/19/2018	neg	na	*Callithrix*	epi	urban	good	Uberlandia	-18,91	-48,27	Triangulo/Alto Paranaíba
694	2/21/2018	pos	11	*Callithrix*	epi	rural	good	Ouro Preto	-20,28	-43,5	Metropolitana
696	2/21/2018	pos	28	*Callithrix*	epi	rural	intermediate	Uberaba	-19,74	-47,93	Triangulo/Alto Paranaíba
687	2/21/2018	neg	na	*Callithrix*	epi	rural	good	Silvianopolis	-22,02	-45,83	Sul/Sudoeste de Minas
688	2/21/2018	neg	na	na	epi	rural	good	Espirito Santo do Dourado	-22,04	-45,95	Sul/Sudoeste de Minas
665	2/21/2018	neg	na	*Callithrix*	epi	urban	good	Belo Horizonte	-19,81	-43,95	Metropolitana

681	2/21/2018	neg	na	*Callithrix*	epi	urban	good	Uberlandia	-18,91	-48,27	Triangulo/Alto Paranaíba
677	2/21/2018	neg	na	*Callithrix*	epi	rural	intermediate	Lontra	-15,9	-44,3	Norte de Minas
703	2/21/2018	neg	na	*Callithrix*	epi	urban	intermediate	Araxa	-19,59	-46,94	Triangulo/Alto Paranaíba
666	2/22/2018	pos	37	*Callithrix*	epi	urban	bad	Buenopolis	-17,87	-44,18	Central Mineira
679	2/22/2018	neg	na	*Callithrix*	epi	rural	bad	Divinesia	-20,99	-43	Zona da Mata
699	2/22/2018	neg	na	*Callithrix*	epi	rural	bad	Uba	-21,12	-42,94	Zona da Mata
664	2/22/2018	neg	na	*Callithrix*	epi	urban	good	Tocantins	-21,17	-43,01	Zona da Mata
692	2/26/2018	neg	na	*Alouatta*	epi	rural	good	Cabeceira Grande	-16,03	-47,09	Noroeste de Minas
671	2/26/2018	neg	na	*Alouatta*	epi	rural	good	Leopoldina	-21,53	-42,64	Zona da Mata
682	2/26/2018	neg	na	*Callithrix*	epi	rural	good	Leopoldina	-21,53	-42,64	Zona da Mata
684	2/26/2018	neg	na	*Callithrix*	epi	urban	good	Belo Horizonte	-19,81	-43,95	Metropolitana
669	2/27/2018	neg	na	*Callithrix*	epi	rural	good	Sao Sebastiao do Paraiso	-20,91	-46,99	Sul/Sudoeste de Minas
685	2/27/2018	neg	na	na	epi	rural	good	Corrego Novo	-19,83	-42,39	Vale do Rio Doce
672	2/27/2018	neg	na	*Callithrix*	epi	urban	good	Antonio Dias	-19,65	-42,87	Zona da Mata
700	2/28/2018	pos	34.4	*Callithrix*	epi	rural	good	Iturama	-19,72	-50,19	Triangulo/Alto Paranaíba
683	2/28/2018	neg	na	na	epi	rural	good	Itapeva	-22,76	-46,22	Sul/Sudoeste de Minas
670	2/28/2018	neg	na	*Callithrix*	epi	urban	good	Divinopolis	-20,13	-44,88	Oeste de Minas
676	2/28/2018	neg	na	*Callithrix*	epi	urban	good	Uberlandia	-18,91	-48,27	Triangulo/Alto Paranaíba
723	3/6/2018	neg	na	*Callithrix*	epi	urban	good	Liberdade	-22,02	-44,32	Sul/Sudoeste de Minas
727	3/6/2018	neg	na	*Callithrix*	epi	urban-rural	good	Divinopolis	-20,13	-44,88	Oeste de Minas
704	3/7/2018	pos	36	*Callithrix*	epi	urban	good	Belo Horizonte	-19,81	-43,95	Metropolitana
730	3/7/2018	neg	na	*Callithrix*	non-epi	na	good	Uberlandia	-18,91	-48,27	Triangulo/Alto Paranaíba
726	3/7/2018	neg	na	*Callithrix*	epi	urban	good	Araguari	-18,64	-48,18	Triangulo/Alto Paranaíba
724	3/7/2018	neg	na	*Callithrix*	epi	urban	good	Coromandel	-18,47	-47,2	Triangulo/Alto Paranaíba

728	3/7/2018	neg	na	*Callithrix*	epi	urban	good	Monte Carmelo	-18,72	-47,49	Triangulo/Alto Paranaíba
707	3/7/2018	neg	na	*Callithrix*	epi	urban	good	Uberlandia	-18,91	-48,27	Triangulo/Alto Paranaíba
725	3/7/2018	neg	na	*Callithrix*	epi	urban	good	Uberlandia	-18,91	-48,27	Triangulo/Alto Paranaíba
720	3/8/2018	neg	na	*Callithrix*	epi	urban	bad	Sete Lagoas	-19,46	-44,24	Metropolitana
721	3/8/2018	neg	na	*Callithrix*	epi	urban	intermediate	Sete Lagoas	-19,46	-44,24	Metropolitana
717	3/9/2018	neg	na	*Callithrix*	epi	urban	good	Divinopolis	-20,13	-44,88	Oeste de Minas
713	3/13/2018	neg	na	*Callithrix*	epi	urban	good	Sao Sebastiao do Paraiso	-20,91	-46,99	Sul/Sudoeste de Minas
711	3/14/2018	pos	35	*Callithrix*	epi	urban	good	Estrela do Sul	-18,74	-47,69	Triangulo/Alto Paranaíba
715	3/14/2018	pos	36.6	*Callithrix*	epi	rural	good	Patrocinio	-18,94	-46,99	Triangulo/Alto Paranaíba
708	3/15/2018	pos	11	*Callithrix*	epi	rural	good	Andrelandia	-21,74	-44,3	Triangulo/Alto Paranaíba
706	3/15/2018	pos	34.5	*Callithrix*	epi	urban	good	Belo Horizonte	-19,81	-43,95	Metropolitana
710	3/16/2018	pos	36.8	*Callithrix*	epi	urban	good	Belo Horizonte	-19,81	-43,95	Metropolitana
714	3/16/2018	neg	na	*Callithrix*	epi	rural	good	Inhauma	-19,49	-44,39	Metropolitana
719	3/21/2018	neg	na	*Callithrix*	epi	urban	intermediate	Arapora	-18,43	-49,18	Triangulo/Alto Paranaíba
742	4/10/2018	neg	na	*Callithrix*	epi	rural	good	Belo Horizonte	-19,81	-43,95	Metropolitana
735	4/10/2018	neg	na	*Callithrix*	epi	urban	good	Passos	-20,71	-46,61	Sul/Sudoeste de Minas
741	4/10/2018	neg	na	*Callithrix*	epi	urban-rural	good	Igaratinga	-19,95	-44,7	Oeste de Minas
747	4/11/2018	pos	12	*Callithrix*	epi	urban-rural	good	Tiradentes	-21,11	-44,17	Campo das Vertentes
740	4/11/2018	neg	na	*Callithrix*	epi	rural	good	Felixlandia	-18,75	-44,89	Central Mineira
734	4/11/2018	neg	na	*Callithrix*	epi	rural	good	Presidente Juscelino	-18,63	-44,05	Central Mineira
745	4/11/2018	neg	na	*Callithrix*	epi	rural	good	Ribeirao das Neves	-19,76	-44,08	Metropolitana
739	4/11/2018	neg	na	*Callithrix*	epi	urban	good	Aracuai	-16,85	-42,07	Jequitinhonha
750	4/11/2018	neg	na	*Callithrix*	epi	urban	good	Sao Romao	-16,36	-45,06	Norte de Minas
732	4/11/2018	neg	na	*Callithrix*	epi	urban	intermediate	Uberaba	-19,74	-47,93	Triangulo/Alto Paranaíba

744	4/12/2018	neg	na	*Callithrix*	epi	urban	good	Belo Horizonte	-19,81	-43,95	Metropolitana
746	4/13/2018	neg	na	*Callithrix*	epi	rural	bad	Monte Carmelo	-18,72	-47,49	Triangulo/Alto Paranaíba
749	4/16/2018	neg	na	*Callithrix*	epi	urban	good	Belo Horizonte	-19,81	-43,95	Metropolitana
736	4/17/2018	neg	na	*Alouatta*	epi	rural	good	Monte Carmelo	-18,72	-47,49	Triangulo/Alto Paranaíba
743	4/17/2018	neg	na	*Callithrix*	epi	urban	good	Belo Horizonte	-19,81	-43,95	Metropolitana
737	4/18/2018	neg	na	*Callithrix*	epi	urban	good	Belo Horizonte	-19,81	-43,95	Metropolitana
738	4/19/2018	neg	na	*Callithrix*	epi	urban	good	Belo Horizonte	-19,81	-43,95	Metropolitana
793	4/23/2018	neg	na	*Callithrix*	epi	urban	good	Muzambinho	-21,37	-46,52	Sul/Sudoeste de Minas
796	4/25/2018	neg	na	*Callithrix*	epi	urban-rural	bad	Itajuba	-22,42	-45,45	Sul/Sudoeste de Minas
775	4/25/2018	neg	na	*Callithrix*	epi	urban-rural	good	Ibirite	-20,02	-44,05	Metropolitana
790	4/26/2018	neg	na	*Callithrix*	epi	urban	intermediate	Visconde do Rio Branco	-21,01	-42,84	Zona da Mata
783	4/26/2018	neg	na	*Callithrix*	epi	urban-rural	intermediate	Guanhaes	-18,77	-42,93	Vale do Rio Doce
782	4/27/2018	neg	na	*Callithrix*	epi	rural	intermediate	Ipatinga	-19,46	-42,53	Vale do Rio Doce
776	4/27/2018	neg	na	*Callithrix*	epi	urban	intermediate	Nova Resende	-21,12	-46,42	Sul/Sudoeste de Minas
774	4/27/2018	neg	na	*Callithrix*	epi	urban	intermediate	Ribeirao das Neves	-19,76	-44,08	Metropolitana
784	5/7/2018	neg	na	*Callithrix*	epi	urban	bad	Leopoldina	-21,53	-42,64	Zona da Mata
761	5/7/2018	neg	na	*Callithrix*	epi	rural	good	Leopoldina	-21,53	-42,64	Zona da Mata
767	5/7/2018	neg	na	*Callithrix*	epi	urban	good	Belo Horizonte	-19,81	-43,95	Metropolitana
777	5/7/2018	neg	na	*Callithrix*	epi	urban	good	Belo Horizonte	-19,81	-43,95	Metropolitana
780	5/7/2018	neg	na	*Callithrix*	epi	urban	good	Belo Horizonte	-19,81	-43,95	Metropolitana
794	5/7/2018	neg	na	*Callithrix*	epi	urban	good	Pirapora	-17,34	-44,94	Norte de Minas
789	5/7/2018	neg	na	*Callithrix*	epi	urban-rural	intermediate	Augusto de Lima	-18,1	-44,26	Central Mineira
751	5/8/2018	neg	na	*Callithrix*	epi	rural	good	Uberaba	-19,74	-47,93	Triangulo/Alto Paranaíba

760	5/8/2018	neg	na	*Callithrix*	epi	rural	good	Uberaba	-19,74	-47,93	Triangulo/Alto Paranaíba
773	5/8/2018	neg	na	*Callithrix*	epi	rural	good	Uberaba	-19,74	-47,93	Triangulo/Alto Paranaíba
762	5/8/2018	neg	na	*Callithrix*	epi	urban	good	Belo Horizonte	-19,81	-43,95	Metropolitana
788	5/8/2018	neg	na	*Callithrix*	epi	urban	good	Contagem	-19,93	-44,05	Metropolitana
787	5/8/2018	neg	na	*Callithrix*	epi	urban	good	Uberaba	-19,74	-47,93	Triangulo/Alto Paranaíba
792	5/8/2018	neg	na	*Callithrix*	epi	rural	intermediate	Arinos	-15,91	-46,1	Noroeste de Minas
755	5/8/2018	neg	na	*Callithrix*	epi	rural	intermediate	Uberaba	-19,74	-47,93	Triangulo/Alto Paranaíba
766	5/8/2018	neg	na	*Callithrix*	epi	rural	intermediate	Uberaba	-19,74	-47,93	Triangulo/Alto Paranaíba
781	5/8/2018	neg	na	*Callithrix*	epi	urban	intermediate	Governador Valadares	-18,85	-41,94	Vale do Rio Doce
778	5/8/2018	neg	na	*Callithrix*	epi	urban	intermediate	Paracatu	-17,22	-46,87	Noroeste de Minas
759	5/9/2018	neg	na	*Callithrix*	epi	urban	good	Monte Santo de Minas	-21,19	-46,98	Sul/Sudoeste de Minas
771	5/9/2018	neg	na	*Callithrix*	epi	urban	good	Uberlandia	-18,91	-48,27	Campo das Vertentes
779	5/9/2018	neg	na	*Callithrix*	epi	urban	good	Uberlandia	-18,91	-48,27	Triangulo/Alto Paranaíba
791	5/9/2018	neg	na	*Callithrix*	epi	urban	intermediate	Sao Tiago	-20,91	-44,5	Campo das Vertentes
752	5/14/2018	neg	na	*Callithrix*	epi	urban	good	Belo Horizonte	-19,81	-43,95	Metropolitana
758	5/14/2018	neg	na	*Callithrix*	epi	urban	good	Belo Horizonte	-19,81	-43,95	Metropolitana
786	5/14/2018	neg	na	*Callithrix*	epi	urban	intermediate	Belo Horizonte	-19,81	-43,95	Metropolitana
785	5/15/2018	neg	na	*Callithrix*	epi	rural	good	Timoteo	-19,58	-42,64	Zona da Mata
769	5/15/2018	neg	na	*Callithrix*	epi	urban	good	Belo Horizonte	-19,81	-43,95	Metropolitana
770	5/15/2018	neg	na	*Callithrix*	epi	urban	good	Tiradentes	-21,11	-44,17	Campo das Vertentes
765	5/16/2018	neg	na	*Callithrix*	epi	rural	good	Prudente de Morais	-19,48	-44,15	Metropolitana
764	5/16/2018	neg	na	*Callithrix*	epi	urban	good	Monte Santo de Minas	-21,19	-46,98	Sul/Sudoeste de Minas
754	5/17/2018	neg	na	*Callithrix*	epi	urban	na	Alem Paraiba	-21,88	-42,7	Zona da Mata
795	5/17/2018	neg	na	na	epi	urban	na	Buenopolis	-17,87	-44,18	Central Mineira
763	5/17/2018	neg	na	na	epi	urban	na	Cataguases	-21,38	-42,69	Zona da Mata

772	5/21/2018	neg	na	*Callithrix*	epi	urban	good	Belo Horizonte	-19,81	-43,95	Metropolitana
757	5/22/2018	neg	na	*Callithrix*	epi	rural	good	Estrela do Sul	-18,74	-47,69	Triangulo/Alto Paranaíba
768	5/22/2018	neg	na	*Callithrix*	epi	urban	intermediate	Governador Valadares	-18,85	-41,94	Vale do Rio Doce
828	5/23/2018	neg	na	*Callithrix*	epi	urban	good	Belo Horizonte	-19,81	-43,95	Metropolitana
800	5/23/2018	neg	na	*Callithrix*	epi	urban	good	Itajuba	-22,42	-45,45	Sul/Sudoeste de Minas
833	5/24/2018	neg	na	*Callithrix*	epi	urban	good	Gouveia	-18,45	-43,74	Jequitinhonha
803	5/29/2018	neg	na	*Callithrix*	epi	urban	bad	Uberlandia	-18,91	-48,27	Triangulo/Alto Paranaíba
812	5/29/2018	neg	na	*Callithrix*	epi	urban	good	Uberlandia	-18,91	-48,27	Triangulo/Alto Paranaíba
826	5/29/2018	neg	na	*Callithrix*	epi	urban	good	Uberlandia	-18,91	-48,27	Triangulo/Alto Paranaíba
801	5/29/2018	neg	na	*Callithrix*	epi	urban	intermediate	Abadia dos Dourados	-18,48	-47,4	Triangulo/Alto Paranaíba
804	5/30/2018	neg	na	*Callithrix*	epi	urban	bad	Cataguases	-21,38	-42,69	Zona da Mata
811	5/30/2018	neg	na	*Callithrix*	epi	rural	good	Uruana de Minas	-16,06	-46,25	Noroeste de Minas
816	5/30/2018	neg	na	*Callithrix*	epi	urban	good	Belo Horizonte	-19,81	-43,95	Metropolitana
819	5/30/2018	neg	na	*Callithrix*	non-epi	rural	intermediate	Uruana de Minas	-16,06	-46,25	Noroeste de Minas
827	6/5/2018	neg	na	*Callithrix*	non-epi	urban	bad	Mantena	-18,78	-40,98	Vale do Rio Doce
807	6/5/2018	neg	na	*Callithrix*	non-epi	rural	good	Tiros	-19	-45,96	Triangulo/Alto Paranaíba
818	6/5/2018	neg	na	*Callithrix*	non-epi	urban	good	Guaxupe	-21,3	-46,71	Sul/Sudoeste de Minas
815	6/5/2018	neg	na	*Callithrix*	non-epi	urban	good	Lagoa Formosa	-18,77	-46,4	Triangulo/Alto Paranaíba
832	6/5/2018	neg	na	*Callithrix*	non-epi	urban	good	Ribeirao das Neves	-19,76	-44,08	Metropolitana
797	6/5/2018	neg	na	*Callithrix*	non-epi	rural	intermediate	Carmo do Paranaiba	-19	-46,31	Triangulo/Alto Paranaíba
820	6/6/2018	neg	na	*Callithrix*	non-epi	rural	good	Arceburgo	-21,36	-46,94	Sul/Sudoeste de Minas
831	6/6/2018	neg	na	*Callithrix*	non-epi	rural	good	Arceburgo	-21,36	-46,94	Sul/Sudoeste de Minas
829	6/6/2018	neg	na	*Callithrix*	non-epi	rural	good	Catas Altas	-20,07	-43,4	Metropolitana
825	6/6/2018	neg	na	*Callithrix*	non-epi	rural	good	Lassance	-17,88	-44,57	Norte de Minas
814	6/6/2018	neg	na	*Callithrix*	non-epi	rural	good	Uberlandia	-18,91	-48,27	Triangulo/Alto Paranaíba

798	6/6/2018	neg	na	na	non-epi	rural	good	Ibituruna	-21,15	-44,74	Oeste de Minas
824	6/6/2018	neg	na	*Callithrix*	non-epi	urban	good	Uberlandia	-18,91	-48,27	Triangulo/Alto Paranaíba
817	6/7/2018	neg	na	*Callithrix*	non-epi	urban	good	Cataguases	-21,38	-42,69	Zona da Mata
799	6/11/2018	neg	na	*Callithrix*	non-epi	urban	good	Belo Horizonte	-19,81	-43,95	Metropolitana
809	6/12/2018	neg	na	*Callithrix*	non-epi	urban	good	Curvelo	-18,75	-44,43	Central Mineira
830	6/13/2018	neg	na	*Callithrix*	non-epi	urban	good	Buenopolis	-17,87	-44,18	Central Mineira
822	6/13/2018	neg	na	*Callithrix*	non-epi	urban	good	Planura	-20,13	-48,7	Triangulo/Alto Paranaíba
805	6/13/2018	neg	na	*Callithrix*	non-epi	rural	intermediate	Frutal	-20,02	-48,94	Triangulo/Alto Paranaíba
821	6/13/2018	neg	na	*Callithrix*	non-epi	urban	intermediate	Araxa	-19,59	-46,94	Triangulo/Alto Paranaíba
834	6/13/2018	neg	na	*Callithrix*	non-epi	urban	intermediate	Araxa	-19,59	-46,94	Triangulo/Alto Paranaíba
813	6/13/2018	neg	na	*Callithrix*	non-epi	urban	intermediate	Sao Sebastiao do Paraiso	-20,91	-46,99	Sul/Sudoeste de Minas
835	6/15/2018	neg	na	*Callithrix*	non-epi	rural	intermediate	Gouveia	-18,45	-43,74	Jequitinhonha
802	6/21/2018	neg	na	*Callithrix*	non-epi	urban	good	Belo Horizonte	-19,81	-43,95	Metropolitana
810	6/21/2018	neg	na	*Callithrix*	non-epi	urban	good	Belo Horizonte	-19,81	-43,95	Metropolitana
823	6/21/2018	neg	na	*Callithrix*	non-epi	urban	good	Curvelo	-18,75	-44,43	Central Mineira
808	6/21/2018	neg	na	*Callithrix*	non-epi	urban	intermediate	Aimores	-19,49	-41,06	Vale do Rio Doce
838	6/25/2018	neg	na	*Callithrix*	non-epi	urban	good	Ibirite	-20,02	-44,05	Metropolitana
845	6/25/2018	neg	na	na	non-epi	urban	good	Sericita	-20,47	-42,48	Zona da Mata
846	6/26/2018	neg	na	*Callithrix*	non-epi	urban	good	Governador Valadares	-18,85	-41,94	Vale do Rio Doce
869	6/27/2018	neg	na	*Callithrix*	non-epi	urban	good	Acucena	-19,07	-42,54	Vale do Rio Doce
848	6/28/2018	neg	na	*Callithrix*	non-epi	urban	good	Guape	-20,76	-45,91	Sul/Sudoeste de Minas
867	7/4/2018	neg	na	*Callithrix*	non-epi	rural	na	Alpinopolis	-20,86	-46,38	Sul/Sudoeste de Minas
840	7/5/2018	neg	na	*Callithrix*	non-epi	rural	na	Sacramento	-19,86	-47,44	Triangulo/Alto Paranaíba
865	7/9/2018	neg	na	*Callithrix*	non-epi	rural	na	Itabira	-19,61	-43,22	Metropolitana
855	7/16/2018	neg	na	*Callithrix*	non-epi	urban	intermediate	Paracatu	-17,22	-46,87	Noroeste de Minas

841	7/18/2018	neg	na	*Callithrix*	non-epi	rural	good	Passos	-20,71	-46,61	Sul/Sudoeste de Minas
837	7/18/2018	neg	na	*Callithrix*	non-epi	urban	good	Arapora	-18,43	-49,18	Triangulo/Alto Paranaíba
857	7/19/2018	neg	na	*Callithrix*	non-epi	urban	good	Belo Horizonte	-19,81	-43,95	Metropolitana
856	7/19/2018	neg	na	*Callithrix*	non-epi	rural	intermediate	Leopoldina	-21,53	-42,64	Zona da Mata
860	7/20/2018	neg	na	*Callithrix*	non-epi	urban	good	Belo Horizonte	-19,81	-43,95	Metropolitana
844	7/24/2018	neg	na	*Callithrix*	non-epi	urban	good	Governador Valadares	-18,85	-41,94	Vale do Rio Doce
864	7/24/2018	neg	na	*Callithrix*	non-epi	urban	good	Governador Valadares	-18,85	-41,94	Vale do Rio Doce
861	7/24/2018	neg	na	*Callithrix*	non-epi	urban	intermediate	Sao Jose da Lapa	-19,7	-43,95	Metropolitana
836	7/24/2018	neg	na	*Alouatta*	non-epi	urban	na	Belo Horizonte	-19,81	-43,95	Metropolitana
862	7/25/2018	neg	na	*Callithrix*	non-epi	rural	bad	Cana Verde	-21,02	-45,18	Oeste de Minas
868	7/25/2018	neg	na	*Callithrix*	non-epi	urban	good	Barra Longa	-20,28	-43,04	Zona da Mata
849	7/26/2018	neg	na	*Callithrix*	non-epi	urban-rural	good	Rio Paranaiba	-19,19	-46,24	Triangulo/Alto Paranaíba
843	7/26/2018	neg	na	*Callithrix*	non-epi	urban	intermediate	Patos de Minas	-18,57	-46,51	Triangulo/Alto Paranaíba
851	8/2/2018	neg	na	*Callithrix*	non-epi	rural	good	Bom Despacho	-19,73	-45,25	Central Mineira
859	8/2/2018	neg	na	*Callithrix*	non-epi	rural	good	Sao Joao Nepomuceno	-21,54	-43,01	Zona da Mata
852	8/3/2018	neg	na	na	non-epi	rural	good	Sao Domingos do Prata	-19,86	-42,96	Metropolitana
854	8/3/2018	neg	na	*Callithrix*	non-epi	urban	good	Conceicao da Aparecida	-21,09	-46,2	Sul/Sudoeste de Minas
839	8/3/2018	neg	na	*Callithrix*	non-epi	rural	intermediate	Pedro Leopoldo	-19,61	-44,04	Metropolitana
863	8/6/2018	neg	na	*Callithrix*	non-epi	urban	good	Lontra	-15,9	-44,3	Norte de Minas
858	8/7/2018	neg	na	*Callithrix*	non-epi	rural	good	Cabeceira Grande	-16,03	-47,09	Noroeste de Minas
842	8/7/2018	neg	na	*Callithrix*	non-epi	urban	good	Sao Sebastiao do Paraiso	-20,91	-46,99	Sul/Sudoeste de Minas
853	8/10/2018	neg	na	*Callithrix*	non-epi	urban	good	Belo Horizonte	-19,81	-43,95	Metropolitana
866	8/16/2018	neg	na	*Callithrix*	non-epi	rural	good	Inhauma	-19,49	-44,39	Metropolitana
882	8/21/2018	neg	na	*Callithrix*	non-epi	urban	good	Araxa	-19,59	-46,94	Triangulo/Alto Paranaíba

884	8/23/2018	neg	na	*Callithrix*	non-epi	urban	good	Belo Horizonte	-19,81	-43,95	Metropolitana
876	8/24/2018	neg	na	*Callithrix*	non-epi	urban	intermediate	Congonhas	-20,5	-43,85	Metropolitana
870	8/28/2018	neg	na	*Callithrix*	non-epi	urban	good	Belo Horizonte	-19,81	-43,95	Metropolitana
881	8/29/2018	neg	na	*Callithrix*	non-epi	urban	good	Estrela do Sul	-18,74	-47,69	Triangulo/Alto Paranaíba
890	8/30/2018	neg	na	*Callithrix*	non-epi	urban	intermediate	Raposos	-19,96	-43,8	Metropolitana
892	8/31/2018	neg	na	*Callithrix*	non-epi	urban	good	Campo Belo	-20,89	-45,27	Oeste de Minas
873	8/31/2018	neg	na	*Callithrix*	non-epi	urban	intermediate	Leopoldina	-21,53	-42,64	Zona da Mata
874	8/31/2018	neg	na	*Callithrix*	non-epi	urban	intermediate	Leopoldina	-21,53	-42,64	Zona da Mata
883	8/31/2018	neg	na	*Callithrix*	non-epi	urban-rural	intermediate	Visconde do Rio Branco	-21,01	-42,84	Zona da Mata
875	9/11/2018	neg	na	*Callithrix*	non-epi	urban	na	Belo Horizonte	-19,81	-43,95	Metropolitana
880	9/12/2018	neg	na	*Callithrix*	non-epi	urban	good	Guape	-20,76	-45,91	Sul/Sudoeste de Minas
891	9/14/2018	neg	na	*Callithrix*	non-epi	rural	good	Sao Joao Del Rei	-21,13	-44,26	Campo das Vertentes
893	9/17/2018	neg	na	na	non-epi	rural	intermediate	Pimenta	-20,48	-45,79	Oeste de Minas
872	9/21/2018	neg	na	*Callithrix*	non-epi	urban	good	Belo Horizonte	-19,81	-43,95	Metropolitana
887	9/21/2018	neg	na	*Callithrix*	non-epi	urban	good	Belo Horizonte	-19,81	-43,95	Metropolitana
871	9/24/2018	neg	na	*Callithrix*	non-epi	urban	good	Itabira	-19,61	-43,22	Metropolitana
888	9/27/2018	neg	na	*Callithrix*	non-epi	urban	intermediate	Sao Goncalo do Rio Abaixo	-19,82	-43,36	Metropolitana
885	10/1/2018	neg	na	*Callithrix*	non-epi	urban	good	Belo Horizonte	-19,81	-43,95	Metropolitana
889	10/1/2018	neg	na	*Callithrix*	non-epi	urban	good	Itabira	-19,61	-43,22	Metropolitana
886	10/1/2018	neg	na	*Callithrix*	non-epi	urban	good	Iturama	-19,72	-50,19	Triangulo/Alto Paranaíba
879	10/1/2018	neg	na	*Callithrix*	non-epi	rural	intermediate	Itapagipe	-19,9	-49,38	Triangulo/Alto Paranaíba
877	10/3/2018	neg	na	*Callithrix*	non-epi	rural	good	Araguari	-18,64	-48,18	Triangulo/Alto Paranaíba
878	10/3/2018	neg	na	*Callithrix*	non-epi	urban	intermediate	Patrocinio	-18,94	-46,99	Triangulo/Alto Paranaíba
896	10/23/2018	neg	na	*Callithrix*	non-epi	urban	good	Bom Jardim de Minas	-21,94	-44,19	Sul/Sudoeste de Minas

897	11/20/2018	neg	na	*Callithrix*	non-epi	urban	intermediate	Belo Horizonte	-19,81	-43,95	Metropolitana
895	11/29/2018	neg	na	*Callithrix*	non-epi	urban	good	Sete Lagoas	-19,46	-44,24	Metropolitana

ID: identification of non-human primate carcasses. Epi week: epidemiological week. Area: sampling area. Urban-rural: urban-rural interface. Lat: latitude. Long: longitude. RTqPCR-YFV: one-step real time polymerase chain reaction for investigation of yellow fever virus RNA, performed using methods described by Domingo and colleagues, 2012 (DOI: 10.1128/JCM.01799-12). neg: negative result, pos: positive. * total RNA obtained from lung sample. Na: not available.
